# Targeted plasma proteomics reveals signatures discriminating COVID-19 from sepsis with pneumonia

**DOI:** 10.1186/s12931-023-02364-y

**Published:** 2023-02-24

**Authors:** Laura M. Palma Medina, Haris Babačić, Majda Dzidic, Åsa Parke, Marina Garcia, Kimia T. Maleki, Christian Unge, Magda Lourda, Egle Kvedaraite, Puran Chen, Jagadeeswara Rao Muvva, Martin Cornillet, Johanna Emgård, Kirsten Moll, Karolinska K. I./K. COVID-19 Study Group, Jakob Michaëlsson, Malin Flodström-Tullberg, Susanna Brighenti, Marcus Buggert, Jenny Mjösberg, Karl-Johan Malmberg, Johan K. Sandberg, Sara Gredmark-Russ, Olav Rooyackers, Mattias Svensson, Benedict J. Chambers, Lars I. Eriksson, Maria Pernemalm, Niklas K. Björkström, Soo Aleman, Hans-Gustaf Ljunggren, Jonas Klingström, Kristoffer Strålin, Anna Norrby-Teglund

**Affiliations:** 1 Center for Infectious Medicine, Department of Medicine Huddinge, Karolinska Institutet, Karolinska University Hospital, Alfred Nobels Allé 8, 141 52, Stockholm, Sweden; 2 Science for Life Laboratory, Department of Oncology and Pathology, Karolinska Institute, Stockholm, Sweden; 3 Department of Medicine Huddinge, Karolinska Institute, Stockholm, Sweden; 4 Department of Infectious Diseases, Karolinska University Hospital, Stockholm, Sweden; 5 Functional Area of Emergency Medicine, Karolinska University Hospital, Stockholm, Sweden; 6 Childhood Cancer Research Unit, Department of Women’s and Children’s Health, Karolinska Institutet, Stockholm, Sweden; 7 Department of Clinical Pathology and Cancer Diagnostics, Karolinska University Hospital, Stockholm, Sweden; 8 The Laboratory for Molecular Infection Medicine Sweden (MIMS), Umeå, Sweden; 9 Department of Perioperative Medicine and Intensive Care, Karolinska University Hospital, Stockholm, Sweden; 10 Division for Anesthesiology and Intensive Care, Department of Clinical Interventions and Technology CLINTEC, Karolinska Institutet, Stockholm, Sweden

**Keywords:** COVID-19, Community acquired pneumonia, Sepsis, Septic shock, Olink proximity extension assays

## Abstract

**Background:**

COVID-19 remains a major public health challenge, requiring the development of tools to improve diagnosis and inform therapeutic decisions. As dysregulated inflammation and coagulation responses have been implicated in the pathophysiology of COVID-19 and sepsis, we studied their plasma proteome profiles to delineate similarities from specific features.

**Methods:**

We measured 276 plasma proteins involved in Inflammation, organ damage, immune response and coagulation in healthy controls, COVID-19 patients during acute and convalescence phase, and sepsis patients; the latter included (i) community-acquired pneumonia (CAP) caused by Influenza, (ii) bacterial CAP, (iii) non-pneumonia sepsis, and (iv) septic shock patients.

**Results:**

We identified a core response to infection consisting of 42 proteins altered in both COVID-19 and sepsis, although higher levels of cytokine storm-associated proteins were evident in sepsis. Furthermore, microbiologic etiology and clinical endotypes were linked to unique signatures. Finally, through machine learning, we identified biomarkers, such as TRIM21, PTN and CASP8, that accurately differentiated COVID-19 from CAP-sepsis with higher accuracy than standard clinical markers.

**Conclusions:**

This study extends the understanding of host responses underlying sepsis and COVID-19, indicating varying disease mechanisms with unique signatures. These diagnostic and severity signatures are candidates for the development of personalized management of COVID-19 and sepsis.

**Supplementary Information:**

The online version contains supplementary material available at 10.1186/s12931-023-02364-y.

## Background

Infections caused by the severe acute respiratory syndrome coronavirus-2 (SARS-CoV-2) emerged in December of 2019 and rapidly evolved into the global coronavirus disease 2019 (COVID-19) pandemic, which to date has led to over 600 million confirmed cases of COVID-19, including over 6.4 million deaths (covid19.who.int). Although most SARS-CoV-2 infections are mild, the infections can develop into life-threatening conditions associated with acute respiratory distress syndrome (ARDS).

COVID-19 was early on reported as a cytokine-storm mediated disease and an aberrant host response has been implicated in severe cases [1]. This pathobiology resembles sepsis; the most recent clinical criteria (sepsis-3) defines it as life-threatening organ dysfunction caused by a dysregulated host response to infection [2]. The underlying responses in sepsis are complex and heterogenous, involving both pro- and anti-inflammatory immune responses that may manifest as a state of hyperinflammation or immunosuppression [3, 4]. The sepsis-associated dysregulated host response is initiated by pathogen-associated molecular patterns and damage-associated molecular patterns released by damaged host cells, resulting in a direct activation of immune and endothelial cells [5]. The activation leads to release of inflammatory mediators that affect not only the immune system but also the central nervous system, cardiovascular system, vascular endothelium, and the immune system, resulting in acute respiratory distress syndrome, acute kidney injury, multiorgan failure, and septic shock[6].

Early reports of COVID-19 showed elevated levels of pro-inflammatory cytokines, e.g., IL1b, TNF and IL6, especially among severe COVID-19 cases, implicating a cytokine-storm process [7]. In addition, comprehensive proteomics analyses of plasma samples collected from COVID-19 patients confirmed the elevation of these markers as well as many others, including factors of the immune-, complement-, and the coagulation-system, which could be linked to severity and specific COVID-19 outcomes [8–13]. In all studies, an aberrant inflammatory response was evident, but the specific markers implicated as predictive classifiers for severity differed to a large extent. These differences likely reflect the heterogenic nature of the COVID-19 patients with respect to comorbidities and severity of infection, as well as technical aspects such as the analytic assays used and time of sample collection.

Considering the apparent similarities between COVID-19 and sepsis regarding the role of a host-mediated pathophysiology, we set out to compare systemic host responses during the acute stages of COVID-19 and sepsis, to capture potential disease-, pathogen-, and organ-specific proteomic profiles. Using targeted proteomics (covering 290 proteins) on samples from well-defined patient cohorts, we compared plasma proteome signatures in COVID-19 and sepsis clinical endotypes. The COVID-19 cohort included patients enrolled during the first wave of the pandemic within the open resource Karolinska KI/K COVID-19 Immune Atlas effort during spring 2020 [14]. This resource provided insight of T-, B-, Natural Killer-, Mucosal associated invariant T-, Innate lymphoid-, mononuclear phagocyte-, and granulocyte-cell immunotypes relevant for COVID-19 protective immunity and immunopathogenesis [15–21]. As comparator sepsis cohorts, we included (i) patients with community acquired pneumonia (CAP) caused by influenza, (ii) CAP caused by bacterial species, (iii) non-pneumonia sepsis (NP sepsis), and (iv) septic shock. The results revealed a shared core host response to infection, as well as unique proteomics signatures related to specific microbiologic etiology and clinical endotypes. Although COVID-19 and sepsis shared a set of core proteins that were deregulated during infection, the levels of most of these inflammatory proteins were more pronounced in sepsis compared to COVID-19. The comprehensive immune atlas resource from the same patients also allowed for correlation analyses of biomarkers to specific immune cell subpopulations implicated in COVID-19 disease severity. In addition, we applied machine learning (ML) to identify potential biomarkers that could accurately discriminate COVID-19 from CAP-sepsis patients.

## Methods

### Patient cohorts

Plasma samples from SARS-Cov-2 infected patients with COVID-19 admitted at the intensive care or high dependency unit (n = 17, severe COVID-19) or the infectious disease clinic (n = 10, moderate COVID-19) at Karolinska University Hospital, Stockholm were collected for this study. Paired convalescent plasma samples from the COVID-19 groups were collected from 17 patients (8 and 9, from the moderate and severe COVID-19 groups, respectively) approximately 4 months after hospital discharge (median = 136 days, range = 89–153 days). Plasma samples from sepsis patients identified and enrolled in the emergency department were also included. As controls, plasma samples from age- and sex-matched SARS-CoV-2 IgG seronegative healthy volunteers (n = 16) were collected on the same days as the acute COVID-19 patients. Inclusion and exclusion criteria for patient enrollment and collected clinical information of the patients are provided below.

The COVID-19 patients were adult SARS-CoV-2 RNA positive patients who were admitted with acute illness at the Karolinska University Hospital, Stockholm, Sweden in April and May 2020, and were treated at the Infectious Diseases and Intensive Care Unit (ICU) Clinics. Patients with oxygen saturation of 90–94% and/or receiving 0.5–3 L/min of oxygen admitted to the Infectious Diseases Clinic were included to represent moderately ill COVID-19 cases. Patients treated at the ICU or high-dependency unit were included as severely ill COVID-19 cases. Exclusion criteria were age ≥ 80 years, current malignancy, or immunomodulatory treatment prior to hospitalization, to minimize immunosuppression from other causes. Corticosteroid therapy at hospital prior to sampling had been given to 2 moderate- and 12 severe- COVID-19 patients. Plasma samples from the acute phase were collected at the day of study enrollment (i.e., 5–24 days after onset of illness and 1–8 days after hospital admission).

CAP, sepsis, and septic shock patients were identified, enrolled, and sampled for plasma within 2 h after arrival at the Emergency Department of Karolinska University Hospital Huddinge in 2017–2019, as they triggered the department´s sepsis alert [22]. The sepsis alert was triggered in patients with clinical signs of infection combined with either (i) at least one of the following: oxygen saturation < 90% despite oxygen supplementation, respiratory rate > 30 per minute, heart rate > 130 per minute, systolic blood pressure < 90 mmHg, or Glasgow Coma Scale < 8; or (ii) blood lactate > 3.2 mmol/L combined with at least one of the following: oxygen saturation < 95% on room air, respiratory rate > 25 per minute, heart rate > 110 per minute, altered mental status, or temperature > 38.5 °C or < 35 °C.

Patients with pulmonary infiltrates and Influenza virus RNA detected in respiratory tract samples, without any bacterial microorganism detected were selected as Influenza CAP patients (n = 11). Patients with pulmonary infiltrates without any virus detected, but with *Streptococcus pneumoniae* (n = 11), *Haemophilus influenzae* (n = 4), or *Staphylococcus aureus* (n = 2) detected in blood culture or lower respiratory tract culture, or *S. pneumoniae* detected in nasopharyngeal culture (n = 1), or *Mycoplasma pneumoniae* (n = 3) detected by specific PCR on respiratory secretions were selected as bacterial CAP patients.

Patients with sepsis with a sequential organ failure assessment (SOFA) score of ≥ 2 within 12 h from arrival at the hospital and an infectious focus not including the lungs were selected as non-pneumonia sepsis patients. In these patients, blood culture was positive for *Escherichia coli* in 10 patients, *Staphylococcus aureus* in 3 patients, Group A Streptococcus in 1 patient, Group B Streptococcus in 3 patients, and Group C Streptococcus in 1 patient.

Finally, patients with infection, total SOFA score of ≥ 2, lactate > 2, and who received vasopressors were selected as septic shock patients (n = 12). These patients had the following foci of infection: lungs (n = 3), urinary tract infection (n = 5), skin-joint infection (n = 2), abdomen (n = 1), and other (n = 1). Blood culture was positive for *E. coli* in 3 patients (one hade multi-bacterial growth), *Streptococcus pyogenes* in 2 patients, and other gram-negative bacteria in 2 patients.

In the SOFA score calculation, registered values of creatinine, bilirubin, and platelet count values from the period 7–90 days prior to admission were used as baseline values. In the total SOFA score at sampling, baseline SOFA points for pathologic values of creatinine, bilirubin, or platelet count were subtracted.

### Plasma sampling

Plasma was collected in whole-blood tubes containing EDTA (ethylenediaminetetraacetic acid). The COVID-19 patients and healthy controls were sampled using ordinary EDTA tubes that were centrifuged within 2 h and plasma was aspirated and frozen in aliquots at − 80 °C. The sepsis patients were sampled using PPT plasma preparation tubes containing EDTA, that were centrifuged within 2 h and then frozen at − 80 °C. The tubes were thawed once for aspiration and refreezing into aliquots at − 80 °C.

### Quantification of soluble factors

Proximity extension assays (PEA) assays (Olink AB, Uppsala, Sweden) were used for the quantification of selected soluble factors in plasma from all cohorts. All samples were measured using three biomarker panels: organ damage (v.3311), immune response (v.3203), and inflammation (v.3022), each of them targeting 92 protein analytes. Samples that had quality control (QC) warning in all three panels were excluded (1 sepsis and 1 COVID-19 patient), and protein measurements with QC warnings were assigned as missing values. Four proteins were included in two of the panels and measured twice: IL6, CCL11, IL10, and IL5; we assigned the mean of the double measurements as NPX value for the corresponding proteins. For further analyses, only analytes with less than 33% of data under the lower limit of detection (left censored) were considered, yielding a total of 193 proteins analyzed. Individual left-censored values from the analytes included in the study were imputed with the lower limit of detection value.

Protein annotation for GO (gene ontology) biological process terms was performed on the STRING platform [23], all terms related to Innate immune-, adaptive immune-, and Inflammatory- responses were grouped together. The association to specific immune cells was determined with the Human Protein Atlas (HPA) [24] data sets for RNA single cell type, RNA blood cell specific, and RNA blood lineage specific.

Coagulation factors were measured in plasma using three different multiplex panels, including the 6-, 4-, and 3-plex human ProcartaPlex panels (Thermofisher). The samples were measured according to the manufacturer’s instructions and the results are shown in arbitrary units (AU) indicating the log2 of the concentration in the samples in relation to reference plasma provided with the kit. In addition, Thrombomodulin and D-dimer were measured using a Multiplex Luminex^®^ assay (R&D Systems, UK). Assays were performed according to the manufacturer’s guidelines and samples were acquired on a Luminex MAGPIX instrument using xPonent 4.0 Software (Luminex).

### Data analysis

For analysis of PEA data, two-tailed student t test was used for two-group comparisons, the test was paired in the acute-convalescent comparison. Statistical analysis of non-parametric data (clinical parameters and protein concentrations from multiplex) was performed with Mann Whitney U test for two group comparisons and Kruskal Wallis coupled with Dunn’s test. Statistical comparison of categorical variables was performed with Fisher test. All p-values were adjusted for multiple-group comparisons with the false discovery rate (FDR).

To account for the effect of confounders, we built a multivariate limma linear model [25] to adjust protein NPX values of COVID-19, CAP-Sepsis, Other Sepsis patients for age (in years), sex, Charlson comorbidity index, and corticosteroid use prior to sampling. The model included no intercept and contrasted the COVID-19 to CAP-Sepsis and COVID-19 to Other-Sepsis status separately, to derive estimated coefficients and standard errors for a corresponding comparison. Finally, moderated two-sided *t* statistic and *F* statistic were calculated for the comparisons based on adjusted log2-Fold change (FC) and empirical Bayes moderation of the standard errors, and the p-values corrected for multiple testing with the FDR.

Based on the protein expression of all 193 proteins, we plotted the 122 samples on a principal component analysis (PCA) plot and further clustered the data with the partitioning around medoids (PAM) algorithm. For PAM, we tested different number of potential clusters k, ranging from k = 2 to the maximum number of samples groups (k = 9), and eventually selected the number of maximum k (k = 4) where the samples had clear separation and the minimum subgroup sample size (n = 8).

All analyses were performed with R (v.4.0.3), in R Studio (v.1.3.959).Correlation analyses were performed with two-tailed non-parametric Spearman test applied on pairwise complete observations using the packages factoextra (v1.0.7), FactoMineR (v2.3), PerformanceAnalytics (v2.0.4), ggplot2 (v3.3.1), gplots (v3.0.4), pheatmap (v1.0.12), vegan (v2.5-6), corrplot (v0.84), lattice (v0.20-41) and latticeExtra (v0.6-29), stats (v4.0.1), and complexheatmap (v2.5.6). The plots were generated with ggplot2(v3.3.6). The package ggpubr (v0.4.0.999) was used for adding statistical significance stars into the plots. The heatmaps with dendrogram integrated were made with pheatmap (v1.0.12).

To build ML models that would accurately classify COVID-19 from CAP-sepsis, we partitioned the dataset (COVID-19 n = 27, CAP-sepsis n = 32) 1,000 times, with random allocation of 75% of the samples to the training & validation dataset (TrnVD) and 25% to the testing dataset (TstD), maintaining the probabilistic distribution of the two conditions in both TrnVD and TstD. On the 1,000 TrnVD, we first ran iterations of random forest (RF) models (R package caret, v6.0-90) for protein selection, using a leave-one-out cross-validation (LOOCV) strategy, with a minimum node size of 3 nodes, 1,000 trees, and Cohen’s kappa as a metric for model training. On the same 1,000 TrnVD, we then ran iterations of logistic regression with lasso regularization (LR-lasso) models (R package glmnet, v4.1-2) for protein selection, using a LOOCV strategy, alpha = 1, selecting minimal lambda for best model in an iteration. We opted for lasso regularization of LR models because it was the best performer of the three modelling approaches, the other two including ridge regression (alpha = 0) and elastic net (EN) regularization (alpha = 0.5); EN had a comparable performance but selected more proteins in the models.

For each iteration of either model on both TrnVD and TstD, we calculated performance metrics: accuracy, F1 score, sensitivity, specificity, positive predictive value, negative predictive value, and Mathew’s correlation coefficient (MCC). MCC is a more robust estimate of accuracy for unbalanced datasets, and it ranges from -1 (extremely low agreement) to 1 (perfect agreement). To be comparable to accuracy estimates, we transformed it to a normalized MCC (nMCC), with a range from 0 to 100%, following the equation: nMCC = (MCC + 1)/2 × 100%. We used the performance metrics on TstD for the final comparison between the RF and LR-lasso models. The performance metrics were plotted on an accuracy radar plot where mean values of accuracy are presented, along with the range and with 95% confidence intervals (CI) (± 1.9685 × SD).

## Results

### The study cohorts

**Fig. 1:**
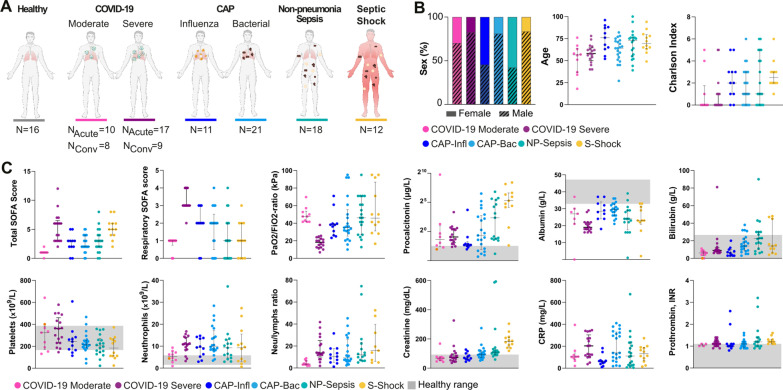
Baseline characteristics of the study cohorts.** A** Number of healthy individuals and patients per group. **B** Distribution of sex, age, and Charlson comorbidity index per group. Colors depict patient subgroups, as indicated. **C** Clinical biomarkers of disease severity at sampling. The grey shadowed areas represent the reference values of the corresponding biomarkers. Significant differences between groups in Additional file 2: Tables S1 and S2. *CAP-Infl* CAP caused by influenza virus, *CAP-Bac* CAP caused by bacteria, *NP-Sepsis* Non-pneumonia sepsis, *S. Shock* Septic shock. *N*_*Acute*_ Number of samples in acute COVID-19. *N*_*Conv*_ Number of samples during convalescence. *NLR* Neutrophil-to-lymphocyte ratio

This study included plasma samples from COVID-19 patients (n = 27 acute and 17 paired convalescent) and sepsis patients (n = 62, all acute), as well as healthy controls (n = 16) to obtain baseline readings (Fig. 1A). COVID-19 patients were enrolled as having either moderate or severe disease based on pre-defined inclusion and exclusion clinical criteria, as detailed in materials and methods. The sepsis patients were classified into different clinical endotypes, i.e., CAP caused by Influenza viruses or bacterial causes, non-pneumonia sepsis, and septic shock. The septic shock cohort includes mostly non-pneumonia cases but also three cases with pneumonia.

Baseline characteristics and laboratory parameters of the study cohorts are shown in Tables 1 and 2, respectively. The patients with severe or moderate COVID-19 did not differ with respect to age, sex, and comorbidities (Table 1; Fig. 1B). However, patients with severe COVID-19 had higher total SOFA score and significantly impaired lung function (e.g., increased respiratory SOFA, decreased PaO_2_/FiO_2_), as compared to patients with moderate COVID-19 (Additional file 2: Table S1; Fig. 1C). In addition, both COVID-19 cohorts displayed abnormal levels of common laboratory markers with the most pathologic levels seen among severe cases (Table 2; Fig. 1C). While lymphocyte and leukocyte counts showed no significant differences between the two COVID-19 severity groups (Additional file 2: Table S1), there was a significant increase of neutrophil counts during severe COVID-19 (Additional file 2: Table S1; Fig. 1C). Compared with the COVID-19 patients, the sepsis cohort patients were older and had higher Charlson comorbidity index (Tables 1; Additional file 2: Table S1; Fig. 1B). Severe COVID-19 and septic shock groups had the highest total SOFA score, while the respiratory SOFA was significantly higher in the severe COVID-19 group as compared to the other groups, including those with pneumonia (Fig. 1C; Additional file 2: Table S1). Additionally, higher levels of creatinine were noted in the non-pneumonia sepsis and septic shock cohorts. This last group also displayed higher levels of Procalcitonin in comparison to the other cohorts (Fig. 1C; Additional file 2: Table S1).

**Table 1:** Patient characteristics, severity parameters, and clinical course of patients with COVID-19 or sepsis

Characteristic	COVID-19		CAP		Other sepsis		Adj. p-value All
	Moderate	Severe	Influenza	Bacterial	NP sepsis	Septic shock	
	(n = 10)	(n = 17)	(n = 11)	(n = 21)	(n = 18)	(n = 12)	
Female sex, No. (%)	3 (30)	3 (17.6)	6 (54.5)	4 (19)	11 (61.1)	2 (17)	*
Age, median (IQR)	57 (41–62)	58 (52–63)	76 (64–88)	64 (54–72)	72 (56–76)	70 (65–79)	*
Charlson comorbidity index, median (IQR)	0 (0–1)	0 (0–1)	2 (1–3)	1 (0–3)	1 (0–4)	3 (2–3)	*
Days symptom debut to admission, median (IQR)	9 (7–9.8)	9 (6–12)	NA	NA	NA	NA	
Days admission to sampling, median (IQR)	4 (2–6.5)	5 (4–7)	0	0	0	0	
Comorbidities, No. (%)							
Heart failure	0	2 (12)	2 (18)	3 (14)	5 (28)	5 (42)	
Ischemic heart disease	2 (20)	1 (6)	2 (18)	3 (14)	2 (11)	2 (17)	
Chronic lung disease	1 (10)	2 (12)	4 (36)	8 (38)	1 (6)	1 (8)	
Diabetes mellitus	3 (30)	5 (29)	4 (36)	5 (24)	5 (28)	4 (33)	
Renal disease	0	1 (6)	1 (9)	3 (14)	3 (17)	3 (25)	
Liver disease	0	0	0	1 (5)	3 (17)	0	
Cerebrovascular disease	0	0	2 (18)	1 (5)	2 (11)	1 (8)	
Dementia	0	1 (6)	0	1 (5)	2 (11)	3 (25)	
Malignancy	1 (10)	0	1 (9)	3 (14)	1 (6)	2 (17)	
Connective tissue disease	1 (10)	0	3 (27)	2 (10)	2 (11)	0	
Severity scores at sampling, median (IQR)							
Total SOFA score^a^	1 (1–1)	6 (3–6)	3 (2–3)	2 (2–4)	3 (2–4)	5 (4–6)	***
Respiratory SOFA score	1 (1–1)	3 (3–4)	2 (2–3)	2(1–2)	1 (0–2)	1 (0–2)	***
PaO_2_/FiO_2_ ratio (kPa)	47.6 (42.0–52.0)	18.3 (12.6–22.4)	38.1 (28.6–39.5)	35 (28.8–50)	48.1 (38.9–68.5)	45.7 (35.8–76.3)	***
Total SOFA ≥ *2*^*b*^, No. (%)	1 (10)	17 (100)	9 (82)	17 (80)	14 (78)	12 (100)	***
Clinical course, No. (%)							
ICU or HDU^c^	0	17 (100)	1 (9)	2 (9.5)	5 (29)	12 (100)	***
Invasive mechanical ventilation^c^	0	13 (92.2)	0	1 (5)	1 (6)	0	***
Vasopressor^c^	0	13 (92.2)	0	0	1 (6)	12 (100)	***
Dead within 28 days	0	4 (23.5)	1 (9)	0	0	1 (8)	*

All p-values are adjusted and refer to Kruskal–Wallis test or Fisher's test. Stars represent significance: *Adj. p-value < 0.05, **Adj. p-value < 0.01, ***Adj. p-value < 0.005. Numerical statistical results are available in Additional file 2: Tables S1 and S2

*ICU* intensive care unit, *HDU* high dependency unit, *CAP* community acquired pneumonia, *NP-Sepsis* non-pneumonia sepsis.

^a^Baseline SOFA score points from pathologic creatinine, bilirubin, and platelet counts were subtracted from the total SOFA score

^b^Four patients with NP-sepsis had a SOFA score of < 2 in the emergency room, but all of them deteriorated to a SOFA score of 2 within 12 h from admission

^c^During hospital stay

**Table 2:** Laboratory parameters at sampling of study groups

Variable median (IQR)	Normal range	COVID-19		CAP		Other sepsis		Adj. p-value All
		Moderate	Severe	Influenza	Bacterial	NP sepsis	Septic shock	
		(n = 10)	(n = 17)	(n = 11)	(n = 21)	(n = 18)	(n = 12)	
C-reactive protein, mg/L	< 3	104 (91–118)	203 (116–273)	60 (43–69)	139 (91–324)	107.5 (29.3–198.3)	131.5 (82.5–184)	*
Procalcitonin, µg/L	< 0.5	0.40 (0.18–1.19)	0.52 (0.30–1.3)	0.2 (0.18–0.23)	0.63 (0.13–5.8)	5.1 (0.43–34)	39.0 (19.5–90)	***
Ferritin, µg/L	10–150	738 (474.5–1442.5)	1832.5 (934–2088)	N.A	N.A	N.A	N.A	N.A
d-dimer, mg/L	< 0.56	0.71 (0.41–0.83)	2.3 (1.45–3.8)	N.A	N.A	N.A	N.A	N.A
Albumin, g/L	33–47	27 (22.5–29)	19 (18–22)	28.5 (24.8–32.8)	29.5 (26–32.3)	24 (20.5–29)	23 (22.3–30.5)	***
Creatinine, µmol/L	< 90	67.5 (55–75.3)	73 (54–92)	70 (61–93.5)	93 (74–117)	108 (93.3–119.8)	182 (154–208.8)	***
Bilirubin, µmol/L	< 26	6 (4–7.8)	9 (8–11)	6 (3–9)	14 (9–22)	22.5 (12.5–30)	14 (8.5–30.5)	***
Platelet count, × 10^9^/L	165–387	324 (190–367)	361 (230–432)	242 (168.5–274.5)	215 (193–251)	204.5 (149.5–257.3)	184 (111.8–262.8)	*
Neutrophil count, × 10^9^/L	1.6–5.9	5.4 (3.9–6.9)	10.9 (8.5–13.8)	9.4 (5.8–13.6)	10.0 (8.4–15.3)	9.2 (5.3–12)	9.2 (3.2–12.6)	
Lymphocyte count, × 10^9^/L	1.1–3.5	1.2 (1–1.78)	0.8 (0.5–0.9)	0.9 (0.75–1.15)	0.9 (0.7–1.4)	0.55 (0.4–0.8)	0.5 (0.3–1.3)	*
Neutrophil to lymphocyte ratio		3.48 (3.04–5.85)	13.67 (11.9–21.8)	10.44 (6.25–15.58)	9.37 (6.42–22.28)	11.5 (7.25–17.22)	15.86 (7.38–32)	**
Leukocyte count, × 10^9^/L	3.5–8.8	7.7 (5.63–9.65)	12.5 (10.4–13.3)	11.6 (7–15.6)	12.0 (10.2–17.9)	11.9 (5.2–13.05)	11.8 (3.6–14.35)	
Prothrombin complex, INR	< 1.2	1.05 (1.0–1.1)	1.1 (1.0–1.2)	1.0 (1.0–1.1)	1.1 (1.0–1.2)	1.2 (1.0–1.4)	1.2 (1.15–1.3)	

*ICU* intensive care unit, *HDU* high dependency unit, *INR* international normalized ratio, *CAP* community acquired pneumonia, *NP-Sepsis* non-pneumonia sepsis, *N.A.* not available

All data is displayed as median levels and IQR in each cohort. All p-values are adjusted and refer to Kruskal–Wallis test. Stars represent significance: *p-value < 0.05, **p-value < 0.01, ***p-value < 0.005. Numerical statistical results are available in Additional file 2: Table S1

### Differential plasma protein profiles reflect the microbial etiology and site of infection

**Fig. 2:**
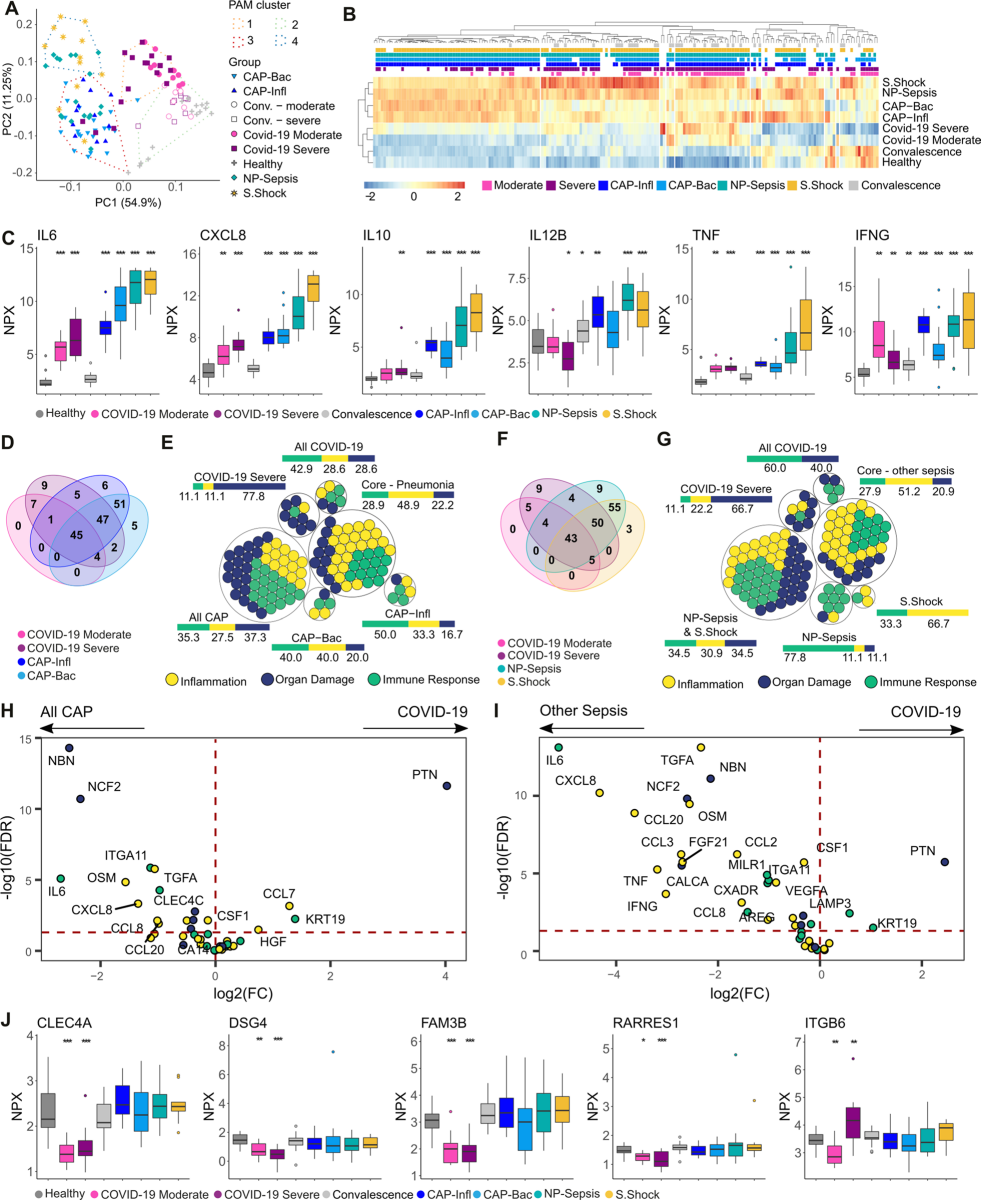
Disease-specific plasma protein signatures in COVID-19 and sepsis. **A** Principal component (PC) analysis based on the levels of all proteins. The PAM clusters are shown by the dashed lines and encompass the samples closest to the cluster’s medoid. **B** Heatmap of mean expression (z scores) of all proteins (x axis) per group with hierarchical clustering (distance: Spearman’s *ρ*). The color-coded boxes denote statistically significant differences in comparison to healthy controls. **C** Plasma levels of classical sepsis-associated cytokines. **D** Venn diagram showing the number of proteins altered in the indicated patient groups compared to healthy controls. **E** Proteins from **D** color-coded based on their PEA panel. The adjacent bars represent the percentage of each PEA panel. Intersections (∩) between groups are denoted as: CAP-Infl ∩ CAP-Bac, “ALL CAP”; severe COVID-19 ∩ moderate COVID-19, “All COVID-19”; all COVID-19 ∩ all CAP, “Core—Pneumonia”; **F** Venn diagram showing the number of proteins altered in the indicated patient groups compared to healthy controls. **G** Proteins from **F** color-coded based on their PEA panel. The adjacent bar represents the percentage of each PEA panel. The “Core—other sepsis” group includes proteins with significantly different levels in the two COVID-19 groups, NP-sepsis, and septic shock; **H**, **I** Volcano plot depicting the difference in plasma levels of the Core-Pneumonia (H) and Core-other sepsis sets (I); color-coded based on the PEA panel. The horizontal dashed line indicates adjusted p values = 0.05; **J** Plasma proteins unique to COVID-19. Boxplots are labeled with gene names and stars represent significance in comparison to healthy controls: *Adj. p-value < 0.05, **Adj. p-value < 0.01, ***Adj. p-value < 0.005. *PAM* partitioning around medoids, *PEA* proximity extension assays

Plasma protein profiles from the patient cohorts and from healthy controls were obtained through PEA using the three Olink 96-targets’ panels for inflammation, immune response, and organ damage. Among the 273 analytes measured, proteins with more than 33% missing values (i.e., under the limit of detection) were excluded, yielding 193 proteins for the final comparative analyses (Additional file 2: Table S3). Unsupervised clustering analyses grouped the patients into four groups, separating acute COVID-19 patients (cluster 1) from healthy controls and convalescent COVID-19 patients (cluster 2), the two CAP groups caused by influenza and bacteria (cluster 3), and septic shock and non-pneumonia sepsis patients (cluster 4) (Fig. 2A). The clearest separation was noticeable on the first principal component, where COVID-19 patients clustered closer to healthy individuals than to sepsis patients. Of note, the cluster dominated by CAP patients (cluster 3) also included three acute COVID patients as well as three septic shock patients; the latter of which all had pneumonia. Hierarchical clustering based on the protein abundance revealed that the separation between the groups could be explained by higher levels of most of the measured proteins in the sepsis cohorts as compared to the COVID-19 patients and healthy controls (Fig. 2B). Severe COVID-19 patients displayed higher levels of proteins than in moderate COVID-19. Although COVID-19 has been reported as a cytokine-storm driven disease [26], the levels of the classical sepsis-associated proteins, i.e., IL6, CXCL8 (Interleukin-8), IL10, IL12, TNF, and IFNγ, did not reach the same magnitude as in CAP-sepsis, non-pneumonia sepsis or septic shock (Fig. 2C). The hierarchical clustering also showed that during convalescence, most proteins normalized and displayed a similar profile to healthy controls (Fig. 2B; Additional file 1: Fig. S1).

To detect alterations of plasma protein levels in each patient cohort, we used as reference the levels in healthy controls. First, we assessed the influence of etiology by comparing the differential expression profiles between patients with lung infections caused by different microbes, i.e., SARS-CoV-2 (COVID-19), Influenza virus (CAP-Infl), or bacteria (CAP-Bac). The results revealed a shared response of 45 proteins, as well as 7, 6, and 5 unique proteins specific for SARS-CoV-2, Influenza, and bacteria, respectively (Fig. 2D; Additional file 2: Table S4). In general, the response profiles, including both shared and unique proteins, showed an equal representation from all three PEA protein panels (inflammation, immune response, and organ damage), except for the set of proteins unique to severe COVID-19, which was dominated by markers of the organ damage response (Fig. 2E). Next, we examined the response profiles in COVID-19 versus other sepsis cases, i.e., non-pneumonia sepsis or septic shock (Fig. 2F). Again, the results revealed a shared core response of 43 proteins with altered abundance, while 5, 9, and 3 were unique to COVID-19, non-pneumonia sepsis and septic shock, respectively. The unique signatures revealed a similar dominance of organ damage proteins in severe COVID-19, while the non-pneumonia sepsis showed a predominance of immune response related proteins (Fig. 2G; Additional file 2: Table S4). The shared core profiles in Fig. 2D, F were essentially identical (42 proteins) and likely depict the basal host response to infection independent of etiology or focus of infection. However, many of the shared factors differed in expression, including several of the pro-inflammatory markers that had substantially higher levels in both CAP-sepsis and other sepsis cohorts, as compared to COVID-19 (Figs. 2H, I; Additional file 2: Table S5). In contrast, only two proteins, pleiotrophin (PTN) and keratin-19 (KRT19), were upregulated in COVID-19. By stratifying the comparison to CAP based on etiology, PTN was consistently higher in COVID-19 compared to both CAP-Infl and CAP-Bac, while KRT19 was only higher when compared to CAP-Bac but not to CAP-Infl (Additional file 1: Fig. S1). Moreover, we observed that the vast majority of these differences remain even after adjustment for the following confounders age, sex, Charlson comorbidity index and the use of corticosteroids prior to sampling (Additional file 1: Fig. S2; Additional file 2: Tables S5, S6).

Taking all patient cohorts into account, a set of five proteins were unique to COVID-19 (Fig. 2J). Among these, four (CLEC4A, DSG4, FAM3B, and RARRES1) displayed significantly lower levels in both moderate and severe COVID-19, as compared to the healthy controls and the sepsis cohorts. ITGB6 had higher levels in severe COVID-19 as compared to all other sepsis cohorts. In contrast, moderate COVID-19 patients had lower ITGB6 levels compared to all other cohorts.

### Plasma biomarkers aid differential diagnosis of COVID-19 and CAP-sepsis

Moderate and severe forms of COVID-19 almost consistently present with pneumonia [27–29], posing as a challenge to differentiate from CAP-sepsis caused by other agents [30, 31]. Therefore, to identify plasma proteins that can serve as biomarkers for accurate differentiation of COVID-19 and CAP-sepsis patients, we employed two ML algorithms, i.e., RF and LR-lasso (Additional file 1: Figure S3).

**Fig. 3:**
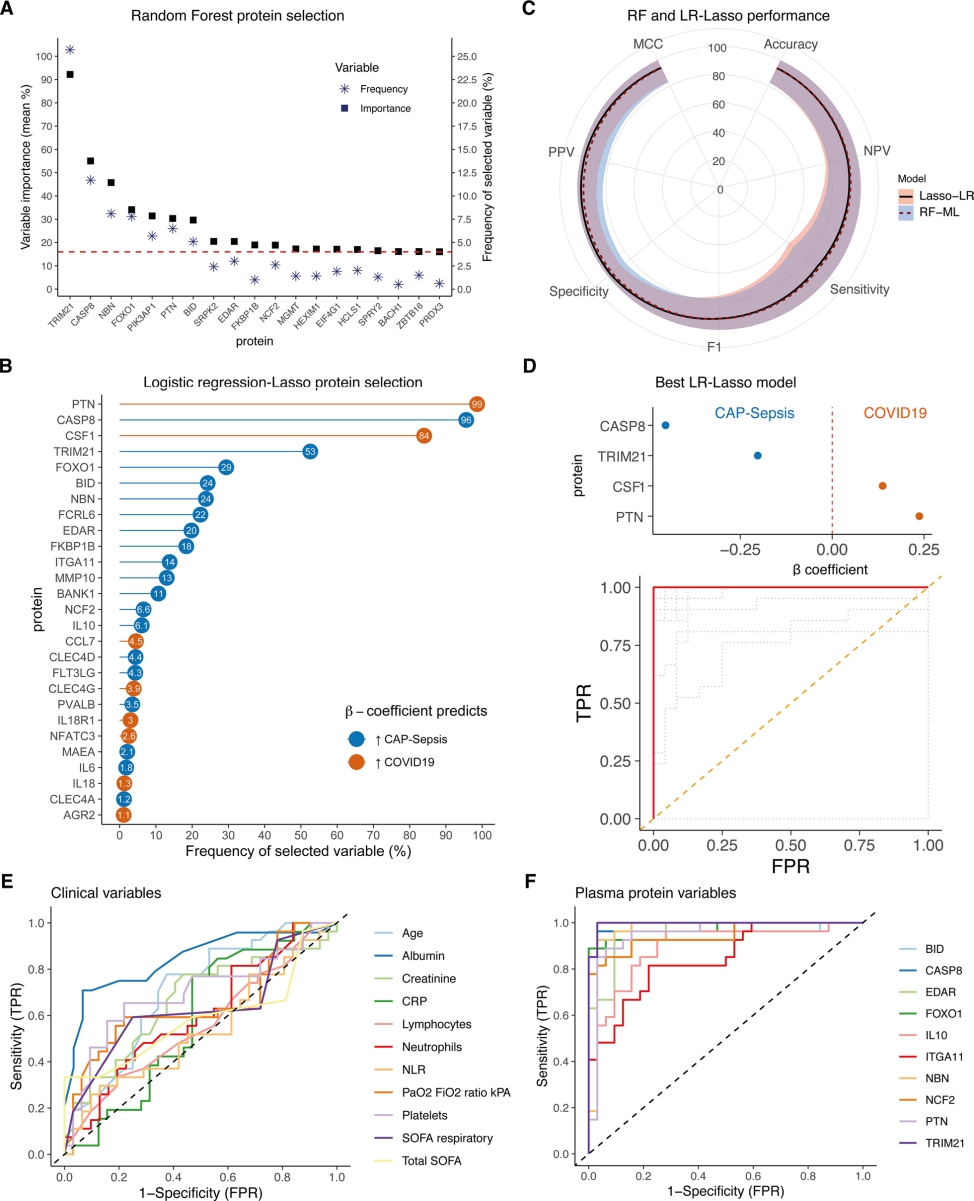
Machine learning models for differentiating COVID-19 from CAP-sepsis. **A** Proteins above the 90th percentile of variable importance (dashed line) selected more frequently in the random forest models (RF-ML). **B** Lollipop plot showing the most frequently selected proteins in the logistic regression models with lasso regularization (LR-Lasso). **C** Accuracy radar plot comparing performance metrics of each model type calculated on testing datasets. The lines represent the mean value of the metric in position and the shadows represent the 95% CI (± 1.97 × SD) of the metric’s mean. **D** The LR-lasso model that had 100% accuracy in both training and testing data, which consisted of the smallest panel of proteins. Colors refer to the β coefficient as in **B**, and the ROC curve shows the model accuracy. The orange dashed line represents chance, the grey dotted lines represent AUCs for different values of lambda. **E** ROC curves demonstrating the diagnostic potential of existing clinical biomarkers in differentiating COVID-19 from CAP-sepsis. The dashed line represents chance. **F** ROC curves for the intersecting most frequently selected proteins in both RF and LR-Lasso models. Additional ROC curves of proteins, see Additional file 1: Fig. S4B, C

Seven proteins, i.e., TRIM21, CASP8, NBN, FOXO1, PIK3AP1, PTN, and BID, had higher average variable importance for the models and were repeatedly selected as biomarkers in the 1000 iterations of the RF models (Fig. 3A). On average, the models had high accuracy in differentiating COVID-19 from CAP on both the training (mean accuracy = 95.01%, range: 91.11–100%) and testing data (mean accuracy = 93.78%, range: 71.43–100%). Four of the five top models with highest accuracy (98% and 100% on training and testing data, respectively), consisted of single proteins TRIM21, PIK3AP1 and NBN, and one model consisted of CASP8 and FLT3LG (Additional file 1: Fig. S4A). In comparison, among the 1000 iterations of the LR-lasso models, four proteins were selected in > 50% of the iterations: PTN, CASP8, CSF1, and TRIM21 (Fig. 3B). Overall, the distribution of performance metrics of the LR-lasso was similar to the RF models, with higher accuracy of LR-lasso models in training data (mean accuracy = 97.24%, range: 93.33–100%, Wilcoxon test: p < 2.2 × 10^–16^) and no difference in testing data (mean accuracy: 93.97%, range: 71.43–100%, Wilcoxon test: p = 0.394). There was a trade-off between slightly better positive predictive value (PPV) and specificity versus worse sensitivity in the LR-lasso models compared to RF models (Fig. 3C, Wilcoxon test: p < 0.05). However, the best LR-lasso models outperformed the best RF models; 93 LR-lasso models had 100% accuracy in both training and testing data, where the algorithms selected from 4 to 17 proteins as predictors in the models. As a final biomarker panel, we selected the LR-lasso model with the smallest panel of plasma proteins that had 100% accuracy in all the metrics, including the AUC (Area Under the Curve) in ROC (Receiver Operating Characteristics) curves (Fig. 3D). This model consisted of four proteins; PTN and CSF1, whose higher levels predicted COVID-19, and TRIM21 and CASP8, whose higher levels predicted CAP.

To assess how the most frequently selected plasma proteins performed as single biomarkers compared to existing clinical variables, we tested their sensitivity and specificity in differentiating COVID-19 from CAP through ROC curves. Among the existing clinical biomarkers, plasma albumin had the best discriminating power (Fig. 3E). Nonetheless, most plasma protein biomarkers that were identified with the two ML algorithms outperformed all the clinical variables in differentiating COVID-19 from CAP, with TRIM21 having the highest AUC (Fig. 3F, see Additional file 1: Fig. S4B, C for ROC curves of the remaining proteins). Single plasma proteins had higher accuracy in differentiating the two conditions than any of the remaining clinical biomarkers, with only four proteins being sufficient to obtain full differentiation between COVID-19 and CAP.

### Host response profiles associated with severity of COVID-19

**Fig. 4:**
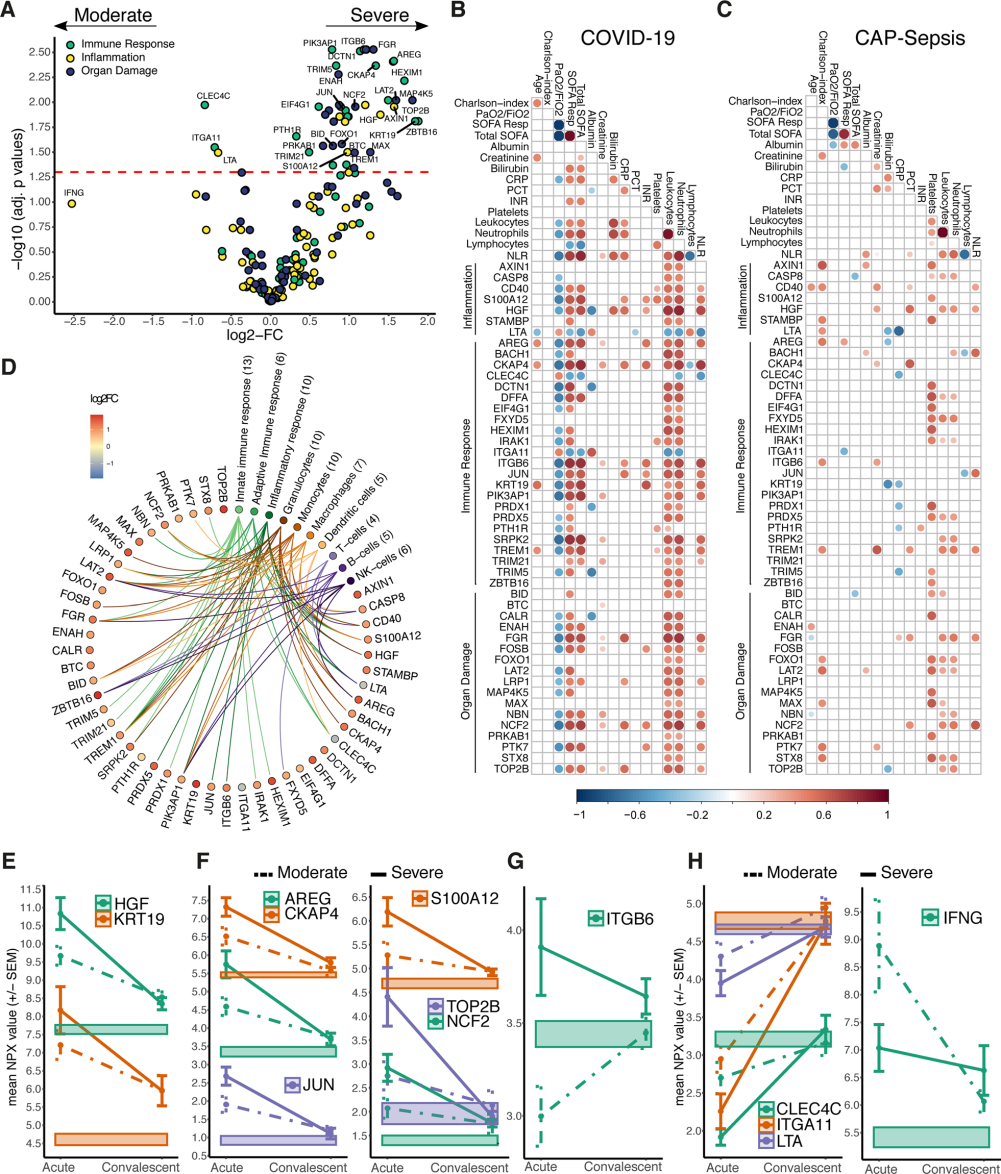
Plasma proteins associated with COVID-19 severity in relation to immune response, clinical variables, and convalescence. **A** Volcano plot depicting the plasma proteome alterations in severe versus moderate COVID-19. The horizontal dashed line indicates the adjusted p values = 0.05. Colors indicate proteins’ PEA panel. **B**, **C** Heatmaps showing statistically significant correlations (Spearman’s, p < 0.05) between the 47 differentially altered plasma proteins in severe COVID-19 and clinical biomarkers of severity in (**B**) COVID-19 patients or (**C**) all CAP-sepsis patients. The bigger circle size and higher colour intensity represent higher correlations. **D** Diagram of the differentially altered plasma proteins in severe COVID-19 annotated by GO terms related to immune responses (based on STRING annotations [23]) and cell types (based on Human Protein Atlas [24]). **E**–**G** Average protein expression (± SEM) during acute and convalescence phases of selected proteins that had higher levels in severe COVID-19 compared to moderate. Proteins were labelled with gene names. **E** The only two proteins that had higher levels in severe COVID-19 in both acute and convalescence phases, as compared to healthy. **F** Proteins correlated (Spearman’s *ρ* > 0.7*)* with both KRT19 and HGF. **G** The only protein among the COVID-19-unique proteins (see Fig. 2J) that was higher in severe COVID-19. **H** Proteins that had lower levels in severe versus moderate (COVID-19)

In comparison to healthy controls, 57 proteins were differentially altered in both COVID-19 groups, regardless of severity, whereas 63 proteins were identified only in severe cases (Additional file 2: Table S4). Most of these markers displayed higher levels than those observed in the healthy controls (Additional file 1: Fig. S5A). Furthermore, a direct comparison of severe to moderate COVID-19 patients showed that among the shared proteins, 44 had higher levels in severe cases, while only 3 proteins had higher levels in moderate cases (Fig. 4A; Additional file 1: Fig. S5B). Most of these proteins correlated with total SOFA, respiratory SOFA score, and PaO_2_/FiO_2_ ratio; as well as with leukocyte and neutrophil counts in the COVID-19 patients (Fig. 4B). In contrast, such associations were not recapitulated in any of the sepsis cohorts (Additional file 1: Fig. S6), not even in those with pulmonary affection*, *i.e., the CAP cohort (Fig. 4C), implicating a particular role of these proteins in the pathophysiology of SARS-CoV-2 infections. To validate our findings, we analyzed a publicly available plasma proteomics dataset on acute phase COVID-19 samples classified according to the WHO severity grades [8]. We compared the WHO severity grades IV and II, which approximate our classification of moderate and severe cohorts, respectively. This dataset included 42 out of the 47 severity-associated proteins. Among these, 27 (64%) recapitulated the statistically significant alterations between the patient groups (Additional file 1: Fig. S7).

To seek further functional insight, we annotated the 47 COVID-19 severity-associated proteins with GO terms, which revealed that the most frequent terms related to innate immune responses and furthermore, that the proteins’ main cellular sources were granulocytes and monocytes (Fig. 4D). Developing on our previous studies using high-dimensional flow cytometry for immunophenotyping of subpopulations of granulocytes and monocytes on our COVID-19 patient cohort [16, 17], we were able to correlate severity-implicated plasma proteins to specific cell surface markers defining immune cell subpopulations. Increased levels of almost all soluble proteins correlated to low expression of activation markers in both granulocyte and monocyte populations, indicating a link between the soluble markers and immature or exhausted innate immune cells in severe COVID-19 (Additional file 1: Fig. S8). In particular, we found an association between plasma proteins elevated in severe COVID-19, such as HGF, AREG, CKAP4, S100A12, NCF2, ITGB6, and a subpopulation of monocytes with lower expression of CD86 and HLA-DR, which are characteristics of myeloid-derived suppressor-like cells (Figure S8A). Likewise, the high levels of most soluble factors correlated with decreased expression of activation markers in neutrophils, e.g., CD16 and CD69, indicating an association with increased frequency of immature CD16^dim^ (Additional file 1: Fig. S8B). Similarly, most soluble factors were inversely correlated to the expression of activation markers in basophils (e.g., CD11b, CD62L and CD177) and eosinophils (e.g., CD66b and CD193), while a positive correlation was observed between the levels of many factors and the activation markers CXCR4 and FceR1 in eosinophils and basophils, respectively.

We further examined the levels of these 47 proteins during convalescent phase four months after acute disease. At this time point, all patients subjected to convalescent sampling had recovered, although some presented with persisting cough. While most proteins associated to severity normalized during convalescence phase (Additional file 1: Fig. S5B), only HGF and KRT19 remained significantly higher as compared to healthy controls (Fig. 4E; Additional file 2: Table S4). Interestingly, a set of six proteins had a similar behavior to KRT19 and HGF during the acute phase of COVID-19, except that they reached healthy levels during convalescence (Fig. 4F). Among the five proteins uniquely upregulated in COVID-19 patients compared with controls (see Fig. 2J), only ITGB6 had higher levels in acute severe patients compared to healthy controls and remained higher during convalescence (Fig. 4G). The three proteins that were lower in COVID-19 cases compared to healthy controls and had the lowest levels in severe cases, i.e*.*, CLEC4C, LTA (Lymphotoxin α), and ITGA11, all normalized during convalescence (Fig. 4H). Although statistically non-significant at 5% FDR, IFNγ showed the largest difference with lower levels in severe compared to moderate cases (log2-FC = − 2.525, p-value = 0.036, adj. p-value = 0.104). Unlike the previous three proteins, IFNγ levels were above the healthy range in both the acute and convalescence phases.

### Changes in the coagulation cascade are more profound during sepsis

Severe infections that trigger a systemic inflammatory response, like sepsis, commonly present coagulopathies [32]. This effect is also seen in patients with COVID-19 admitted into intensive care, who frequently present with thrombotic complications. Since it has been reported that the coagulation abnormalities presented in COVID-19 patients differ from those in patients with sepsis or trauma [33], we extended the plasma profiling to 14 coagulation factors assessed by Luminex® multiplex.

**Fig. 5:**
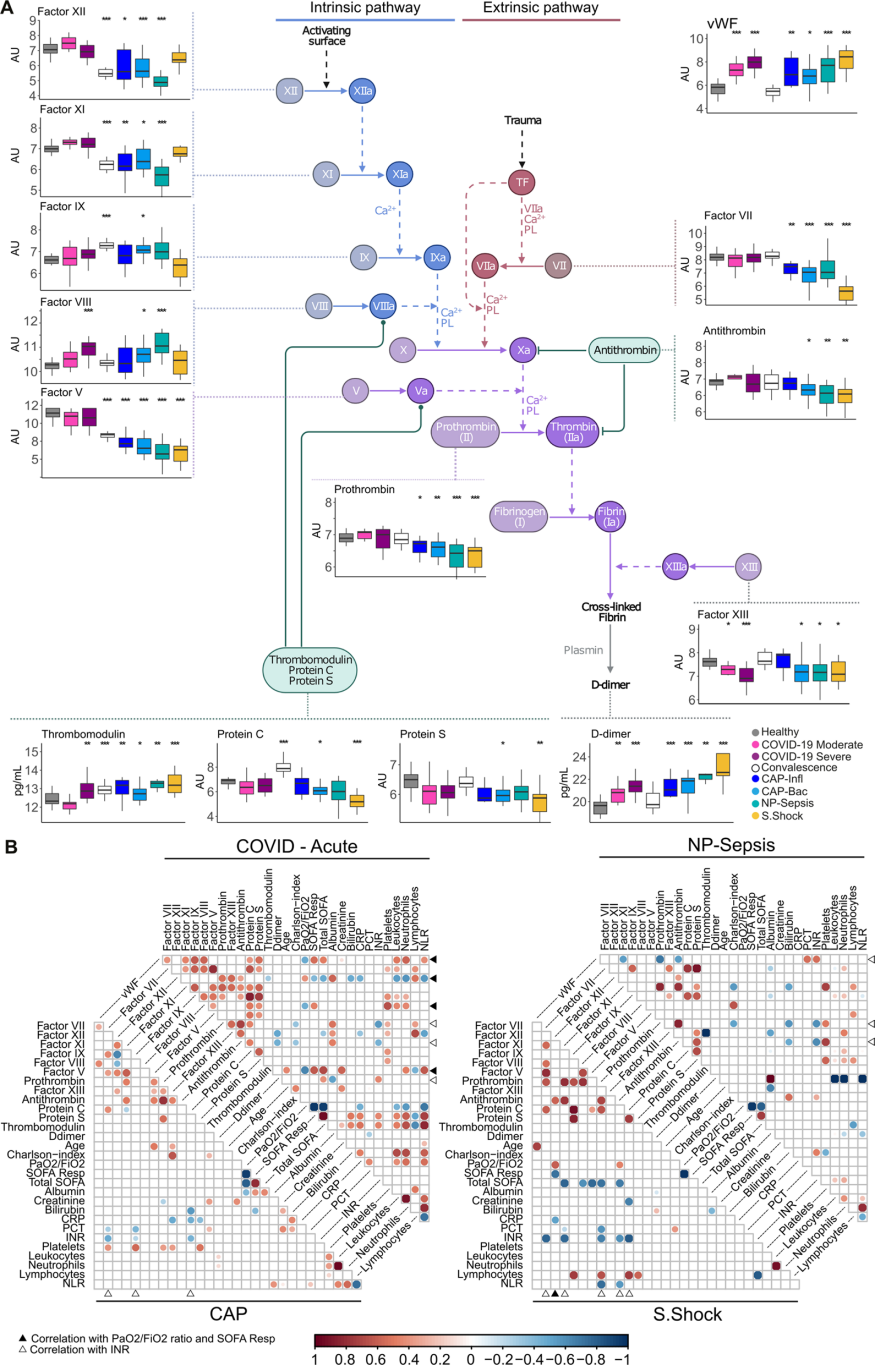
Coagulation cascade-related proteins altered in COVID-19 and sepsis.** A** Coagulation cascade diagram displaying associated protein levels, boxplots are labeled with protein names and stars represent significance in comparison to healthy controls: *Adj. p-value < 0.05, **Adj. p-value < 0.01, ***Adj. p-value < 0.005. **B** Heatmap showing statistically significant correlations (Spearman’s ρ, Adj. p-value < 0.05) between clinical characteristics and coagulation-related proteins. The bigger circle size and higher colour intensity represent higher correlations. The arrows indicate correlation of a coagulation protein with SOFA respiratory and PaO_2_/FiO_2_ ratio (black), or INR (white). The coagulation cascade sketch was adapted from BioRender.com (2022), https://app.biorender.com/biorender-templates. *AU *arbitrary units

In our cohorts, there were no significant differences in prothrombin time (INR) and platelet counts as compared to reference levels (Fig. 1C), not even in three out of the four severe COVID-19 cases with reported thromboembolic events. The Luminex^®^ d-dimer measurements in our patient cohorts were significantly higher compared to healthy controls, but no differences were observed between patients with pulmonary infections of different etiology (COVID-19 vs. CAP). The highest concentrations of D-dimer were measured in septic shock patients and their levels were only significantly higher in comparison to COVID-19 patients, but not to other septic cohorts (Fig. 5A; Additional file 2: Tables S7, S8).

When comparing COVID-19 cases to healthy controls, significant differences were observed in the von Willebrand factor (vWF) and factor XIII levels. Additionally, severe COVID-19 cases also displayed elevated levels of factor VIII and thrombomodulin. All these factors, except thrombomodulin, returned to normal levels in the convalescence phase (Fig. 5A). In contrast, sepsis cases showed more profound changes in the coagulation pathway. Plasma samples from all sepsis cases also displayed an increase in vWF concentration as well as differences in the concentrations of factors VII, V, prothrombin, and thrombomodulin (Fig. 5A). Moreover, differences in factors XII and XI were also noted for CAP and NP patients. Like COVID-19, lower levels of factor XIII were found in CAP-Bac, NP sepsis and septic shock. When comparing the concentrations of antithrombin, protein C and protein S, which regulate the coagulation cascade, we observed that COVID-19 and influenza pneumonia patients did not show significant differences in the concentration of these proteins. In contrast, patients with CAP bacteria and NP sepsis had lower levels of all three of them (Fig. 5A; Additional file 2: Tables S7, S8).

Assessing coagulation factors to clinical markers of severity, we observed that in COVID-19 patients, vWF, XIII, VIII, and thrombomodulin levels correlated with lung function impairment (defined by SOFA respiratory score), whereas such correlation was not seen in the other sepsis cohorts (Fig. 5B). Although our study does not directly conclude on the incidence of coagulopathies in either COVID-19 or sepsis patients, possible tissue damage could have contributed to changes observed in the coagulation cascade, where all proteins altered during COVID-19 (vWF, thrombomodulin, Factor XIII and Factor VIII) are linked to injury and wound repair [34–36]. Overall, these results indicate that the pathophysiology underlying the coagulation abnormalities in COVID-19 may differ from those in sepsis.

## Discussion

In this comparative targeted-proteomics study, we analyzed proteins related to immune-response dysregulation, inflammation, and organ damage in COVID-19 and different sepsis subgroups. As expected, plasma proteome disturbances in both diseases indicated a skewed immune response, hyperinflammation, and organ damage. We demonstrate that COVID-19 and sepsis share a core host response to infection, consisting of 42 plasma proteins that were differentially altered in all infected patient cohorts as compared to healthy controls. Although shared, there was a striking difference in the magnitude of response between the cohorts, with sepsis patients displaying higher levels of most proteins. Among them, several of the classical inflammatory markers, *e.g.*, IL6, IL8, IL10, IL12B, TNF, and IFNγ had substantially higher levels in sepsis compared to COVID-19, leading to the conclusion that the inflammatory response is more pronounced in sepsis regardless of etiology or focus of infection. In addition, the plasma proteome alterations identified unique features associated with respective disease, allowing the discovery of potential plasma biomarkers for differential diagnosis of COVID-19 and CAP-sepsis, among them TRIM21, PTN and CASP8.

Very few proteins had higher levels in COVID-19 compared to sepsis in this study, among them PTN and KRT19. Higher levels of PTN were observed in both COVID-19 cohorts compared to healthy controls and other sepsis cohorts, excluding septic shock. PTN levels were not different between severe and moderate COVID-19; a finding recapitulated in our re-analysis of the data from Filbin et al*.* [8]. However, Filbin et al. reported no difference in PTN between COVID-19 patients and PCR-negative hospital controls, likely due to the heterogeneous selection of controls. Of note, PTN has been reported as a multifunctional cytokine with potential role in inflammation, leukocyte recruitment and tissue regeneration [37]. In accordance with our findings, Filbin et al*.* found higher levels of KRT19 in severe COVID-19 (grade II) as compared to moderate (grade IV) COVID-19. We further showed that KRT19 had higher levels in COVID-19 as compared to CAP-Bac and NP-Sepsis, but similar levels compared to CAP-Infl and septic shock. In line with a previous report suggesting that KRT19 is involved in ARDS-related lung epithelial damage [38], it is tempting to assume that elevated circulating levels of KRT19 are a result of viral-elicited lung tissue injury. It was interesting to observe that both severe and moderate COVID-19 patients had elevated KRT19 even four months after acute disease, suggesting a lingering release of the protein into the bloodstream after lung damage. Similarly, hepatocyte growth factor (HGF), a protein involved in tissue regeneration after damage [39–41], had higher levels in severe COVID-19 during acute phase that persisted during convalescence, indicating its release during tissue repair. This is in line with a previous report implicating HGF in COVID-19 disease severity [42]. This dynamic of protein levels in acute and convalescent phase was specific to KRT19 and HGF, as most proteins upregulated in severe COVID-19 during acute phase normalized during convalescence. Thus, it could be relevant to prospectively monitor KRT19 and HGF in COVID-19, to evaluate their potential value in prognosis of lung impairment post-COVID-19.

We identified a set of 47 proteins dysregulated in severe COVID-19 that were also associated with clinical parameters of disease severity. Among these proteins, KRT19, TOP2B, AREG, HGF, CKAP4, ITGB6, and NCF2 had a higher expression (> twofold) in plasma samples of severe compared to moderate COVID-19 patients, whereas CLEC4C and LTA were among the few proteins that were expressed at lower levels in severe patients. We reproduced these findings in a reanalysis of a public COVID-19 PEA dataset [8]. CLEC4C is of interest as it is a factor linked to anti-viral responses and similarly, low *clec*4C expression has been linked to a particular COVID-19 severity-interaction expression quantitative trait loci [43]. Also, LTA have been reported to be linked to anti-viral responses, i.e. interferon-stimulated genes, and COVID-19 severity [44]. Furthermore, we demonstrated that these proteins correlated with clinical severity scores, such as total SOFA, respiratory SOFA and PaO_2_/FiO_2_ ratio, in COVID-19 patients, whereas no such correlation was observed in any of the sepsis cohorts, not even in CAP patients. Notably, we found a similar association between respiratory dysfunction and the coagulation response, in that several markers, i.e., vWF, Thrombomodulin, Factor XII, and Factor VIII; correlated with respiratory SOFA and PaO_2_/FiO_2_ ratio solely in COVID-19 patients. Taken together, these results underscore important differences in the molecular systemic responses driving the pathophysiology of COVID-19 and CAP-sepsis.

Further analysis of the 47 COVID-19 severity-associated proteins showed strong linkage to monocytes and granulocytes. Utilizing the detailed immunophenotyping published on the same patient cohort [16, 17], allowed for correlation analyses between soluble markers and specific immune cell subpopulations. Several plasma proteins, including HGF, AREG, CKAP4, S100A12, NCF2, and ITGB6, correlated with low expression of CD86 and HLA-DR in all subpopulations of monocytes. In a report by Kvedaraite et al*.* [16], these cell populations express a myeloid-derived suppressor cells-like phenotype that were enriched in severe COVID-19 cases. Likewise, plasma proteins in severe COVID-19 were associated with reduced activation makers of different granulocyte subpopulations, including neutrophils, basophils and most notably eosinophils, which have been reported by Lourda et al*.* [17] to be elevated in severe COVID-19 cases. Taken together, these results show an association between a distinct set of plasma proteins and immature myeloid cell subpopulations reported as elevated in severe COVID-19 disease [16, 17]. These findings are in line with reports pointing to myeloid cells, in particular their immature forms, as important contributors to cytokine-rich environment in COVID-19 [45, 46]. Yet, it is difficult to infer whether these processes are simply conjoined or whether the plasma proteins’ dysregulation precedes the impaired innate immune cell response.

Finally, in light of the observed differences in plasma proteins between COVID-19 and CAP-sepsis, we sought to identify biomarkers with potential use in clinical practice, differentiating the two conditions with similar presentation to aid prompt diagnosis. Although clinical examination, radiological imaging, real-time polymerase-chain reaction (RT-PCR) and bacterial cultures are helpful in differentially diagnosing COVID-19 from CAP-Sepsis, the diagnosis can sometimes be challenging due to inconclusive clinical presentation and/or radiological exams [30], false negative SARS-CoV-2 RT-PCR results [29], or false negative bacterial cultures [3]. This poses as a clinical differential diagnosis problem. Using machine learning, we identified a set of diagnostic plasma biomarkers (e.g., TRIM21, CASP8, PTN and CSF1) that had very high accuracy in differentiating COVID-19 from CAP, and outperformed standard laboratory parameters used in clinical practice. Although some of the models showed perfect accuracy, it is likely overestimated due to the small sample sizes of the two cohorts. One should consider that our findings might be confounded by the different stages of disease, difference in some patient characteristics, and different sampling time periods between the COVID-19 and the sepsis cohorts. The latter also introduces a potential confounder linked to treatment such as corticosteroid use. Even though corticosteroids have been shown to alter blood protein levels in COVID-19 [47, 48] our findings of a more pronounced inflammatory response in CAP-sepsis versus COVID-19 patients were consistent even when adjusting for corticosteroid use and other confounders. Furthermore, the identified potential biomarkers for differential diagnosis in the ML models remained significant in the adjusted multivariate linear models.

One strength of our study design is the inclusion of a prospective sepsis cohort with detailed clinical information allowing for classification into specific clinical endotypes such as CAP caused by Influenza or bacterial causes. We opted for a sepsis cohort enrolled prior to pandemic onset, to ensure that these patients did not have COVID-19, with a limitation in using different sample collection tubes. Both tubes contained EDTA, but the procedure differed as the sepsis samples were collected in PPT tubes (see methods). Although we demonstrate that there are notable differences in plasma protein levels in COVID-19 compared to sepsis, and that they can serve as biomarkers with high accuracy, our findings must be validated in future studies, using larger cohorts. These studies should include cohorts balanced for different clinical characteristics and using samples collected in parallel in the clinical setting.

In this study, we demonstrate that the systemic inflammatory response is higher in sepsis patient as compared to COVID-19 patients. Similar observations have been reported previously using specific sepsis groups, such as bacterial sepsis ARDS or Influenza sepsis [12, 49–52]. Here we show that this difference is observable regardless of microbiologic etiology, site of infection, or septic shock development. In severe COVID-19, immunosuppressive therapy with corticosteroids, interleukin inhibitors, and Janus kinase inhibitors have been shown to improve survival [53–55]. However, corticosteroid therapy may be harmful in the subgroup of hospitalized COVID-19 patients who do not require oxygen therapy[53, 56]. Considering our finding that the inflammatory response was more prominent in sepsis, a greater therapeutic effect of anti-inflammatory agents in sepsis could be expected. However, previous clinical trials showed modest to no clinical efficacy of corticosteroids, interleukin inhibitors, and other anti-inflammatory drugs in sepsis [3, 57–59]. Recent understandings of the high heterogeneity in the sepsis cohorts, including the inter-individual difference in systemic biological host responses to infection, may explain the lack of effect at a group level, highlighting the need for personalized medicine. For example, in septic shock, corticosteroid therapy was recently found to decrease survival in a particular patient subgroup defined by a specific whole-blood transcriptomic signature [60]. Contrary, monocytic HLA-DR-guided immunostimulatory therapy with CSF2 (Granulocyte–macrophage colony-stimulating factor) [61] or IFNγ [62], have shown promising results in patients with severe sepsis. Recently, IFNγ therapy was followed by clinical improvement in five critically ill COVID-19 patients with bacterial complications [63]. This is interesting in light of the low IFNγ found in COVID-19 patients in our study, particularly in the severe group, where six patients had secondary bacterial complications. Thus, identification of circulating biomarkers reflecting endotype-specific disease traits could enable tailored immunomodulatory therapy in sepsis and perhaps also in protracted severe COVID-19.

## Conclusions

Our comparative targeted plasma proteomics study allowed profiling of COVID-19 systemic responses in the context of other severe infectious diseases. The results extend the understanding of the dysregulated host responses underlying severe infections, indicating varying disease mechanisms and hence, the potential of plasma protein signatures as diagnostic tools. Key findings include a shared core response to infection with a skewed immune response, hyperinflammation, and organ damage. While a more pronounced cytokine storm was measured on sepsis, respiratory dysfunction in COVID-19 was linked to a plasma protein signature characterized by markers of tissue damage and wound repair. Furthermore, we used machine learning to pinpoint a set of biomarkers that could accurately discriminate COVID-19 from CAP-sepsis. Such signatures could have potential diagnostic value for management of patients without any positive microbiological results. Overall, the results emphasize the need for personalized medicine in these severe infections and present interesting biomarker candidates for further validation, with the goal of improving the management of COVID-19 and sepsis.

## Supplementary Information

**Additional file 1: Figure S1**. Boxplots of plasma protein levels measured through Proximity Extension Assay. **Figure S2**. Comparison of protein levels between COVID-19 and Sepsis including adjustment for confounders. **Figure S3**. Schematic depiction of the machine learning algorithms used to identify biomarkers for differentiating COVID-19 from CAP-Sepsis. **Figure S4**. Complementary results from machine learning algorithms. **Figure S5**. Comparative analysis of changes in proteins levels occurring in COVID-19 during acute and convalescence phase. **Figure S6**. Heatmap showing correlations between the 47 differentially altered plasma proteins in severe COVID-19 and clinical biomarkers. **Figure S7**. Boxplots of reanalysis of publicly available data provided by Filbin et al. (2021). of the 42 proteins related to COVID-19 severity in our study. **Figure S8**. Heatmaps of correlations between the 47 proteins related to severity of COVID-19 and cellular markers of monocytes and granulocytes. The Karolinska KI/K COVID-19 Study Group

**Additional file 2: Table S1**. Statistical comparisons of clinical data. **Table S2**. Statistical comparisons of patient characteristics. **Table S3**. NPX values of proteins measured by PEA. **Table S4**. Statistical comparisons of protein plasma levels between the cohorts and healthy. **Table S5**. Statistical comparison and fold change of protein levels between COVID-19, all CAP and other Sepsis groups. **Table S6**. Coefficients of limma model comparing protein levels of COVID-19 and Sepsis cohorts adjusting for confounders. **Table S7**. Levels of plasma proteins part of the coagulation cascade. **Table S8**. Statistical comparisons of protein plasma levels in the coagulation cascade between all cohorts.

## Data Availability

All data is available in Additional file 2: Tables S3 and S5 of this manuscript.

## Abbreviations

SARS-CoV-2: Severe acute respiratory syndrome coronavirus-2
COVID-19: Coronavirus disease 2019
ARDS: Acute respiratory distress syndrome
IL1b: Interleukin 1 beta
TNF: Tumor necrosis factor
IL6: Interleukin 6
CAP: Community acquired pneumonia
CAP-Bac: CAP caused by bacterial species
CAP-Infl: CAP caused by influenza
NP-sepsis: Non-pneumonia sepsis
ICU: Intensive Care Unit
SOFA: Sequential organ failure assessment
EDTA: Ethylenediaminetetraacetic acid
PEA: Proximity extension assays
QC: Quality control
HPA: Human Protein Atlas
AU: Arbitrary units
ANOVA: Analysis of variance
FDR: False discovery rate
FC: Fold change
PCA: Principal component analysis
PAM: Partitioning around medoids
TrnVD: Training and validation dataset
TstD: Testing dataset
LOOCV: Leave-one-out cross-validation
RF: Random forest
LR-lasso: Logistic regression with lasso regularization
EN: Elastic net regularization
MCC: Mathew’s correlation coefficient
nMCC: Normalized Mathew’s correlation coefficient
CI: Confidence intervals
CXCL8: Interleukin 8
PTN: Pleiotrophin
KRT19: Keratin-19
PPV: Positive predictive value
AUC: Area under the curve
ROC: Receiver operating characteristics
ML: Machine learning
GO: Gene ontology
LTA: Lymphotoxin α
vWF: Von Willebrand factor
HGF: Hepatocyte growth factor
RT-PCR: Real-time polymerase-chain reaction
CSF2: Granulocyte–macrophage colony-stimulating factor

## References

[1] Osuchowski MF, Winkler MS, Skirecki T, Cajander S, Shankar-Hari M, Lachmann G (2021). The COVID-19 puzzle: deciphering pathophysiology and phenotypes of a new disease entity. Lancet Respir Med.

[2] Bateman RM, Sharpe MD, Jagger JE, Ellis CG, Solé-Violán J, López-Rodríguez M, et al. 36th International Symposium on Intensive Care and Emergency Medicine : Brussels, Belgium. 15–18 March 2016. Crit Care. 2016. p. 94. 10.1186/s13054-016-1208-6 PMC5493079 27885969

[3] Hotchkiss RS, Moldawer LL, Opal SM, Reinhart K, Turnbull IR, Vincent J-L (2016). Sepsis and septic shock. Nat Rev Dis Prim.

[4] Gyawali B, Ramakrishna K, Dhamoon AS (2019). Sepsis: the evolution in definition, pathophysiology, and management. SAGE Open Med.

[5] Cavaillon J-M, Singer M, Skirecki T (2020). Sepsis therapies: learning from 30 years of failure of translational research to propose new leads. EMBO Mol Med.

[6] Gupta A, Madhavan MV, Sehgal K, Nair N, Mahajan S, Sehrawat TS (2020). Extrapulmonary manifestations of COVID-19. Nat Med United States.

[7] Del Valle DM, Kim-Schulze S, Huang H-H, Beckmann ND, Nirenberg S, Wang B (2020). An inflammatory cytokine signature predicts COVID-19 severity and survival. Nat Med.

[8] Filbin MR, Mehta A, Schneider AM, Kays KR, Guess JR, Gentili M (2021). Longitudinal proteomic analysis of severe COVID-19 reveals survival-associated signatures, tissue-specific cell death, and cell–cell interactions. Cell Reports Med..

[9] Messner CB, Demichev V, Wendisch D, Michalick L, White M, Freiwald A (2020). Ultra-high-throughput clinical proteomics reveals classifiers of COVID-19 infection. Cell Syst.

[10] Shen B, Yi X, Sun Y, Bi X, Du J, Zhang C (2020). Proteomic and metabolomic characterization of COVID-19 patient sera. Cell.

[11] Shu T, Ning W, Wu D, Xu J, Han Q, Huang M (2020). Plasma proteomics identify biomarkers and pathogenesis of COVID-19. Immunity.

[12] ConsortiumCOvid-19 Multi-omics Blood ATlas (COMBAT). A blood atlas of COVID-19 defines hallmarks of disease severity and specificity. Cell. 2022;185:916–938.e58. 10.1016/j.cell.2022.01.012 PMC8776501 35216673

[13] Al-Nesf MAY, Abdesselem HB, Bensmail I, Ibrahim S, Saeed WAH, Mohammed SSI (2022). Prognostic tools and candidate drugs based on plasma proteomics of patients with severe COVID-19 complications. Nat Commun.

[14] Ljunggren H-G, Ask EH, Cornillet M, Strunz B, Chen P, Rao Muvva J (2022). The Karolinska KI/K COVID-19 Immune Atlas: an open resource for immunological research and educational purposes. Scand J Immunol.

[15] García M, Kokkinou E, García AC, Parrot T, Medina LMP, Maleki KT (2020). Innate lymphoid cell composition associates with COVID-19 disease severity. Clin Transl Immunol..

[16] Kvedaraite E, Hertwig L, Sinha I, Ponzetta A, Myrberg IH, Lourda M, et al. Major alterations in the mononuclear phagocyte landscape associated with COVID-19 severity. Proc Natl Acad Sci. 2021;118. 10.1073/pnas.2018587118 PMC8017719 33479167

[17] Lourda M, Dzidic M, Hertwig L, Bergsten H, Medina LMP, Sinha I, et al. High-dimensional profiling reveals phenotypic heterogeneity and disease-specific alterations of granulocytes in COVID-19. Proc Natl Acad Sci. 2021;118. 10.1073/pnas.2109123118 PMC8501786 34548411

[18] Maucourant C, Filipovic I, Ponzetta A, Aleman S, Cornillet M, Hertwig L, et al. Natural killer cell immunotypes related to COVID-19 disease severity. Sci Immunol. 2020;5. Available from: https://immunology.sciencemag.org/content/archive/5/50/eabd6832/1. 10.1126/sciimmunol.abd6832 PMC7665314 32826343

[19] Parrot T, Gorin J-B, Ponzetta A, Maleki KT, Kammann T, Emgård J, et al. MAIT cell activation and dynamics associated with COVID-19 disease severity. Sci Immunol. 2020;5. Available from: https://immunology.sciencemag.org/content/5/51/eabe1670. 10.1126/sciimmunol.abe1670 PMC7857393 32989174

[20] Sandberg JT, Varnaitė R, Christ W, Chen P, Muvva JR, Maleki KT (2021). SARS-CoV-2-specific humoral and cellular immunity persists through 9 months irrespective of COVID-19 severity at hospitalisation. Clin Transl Immunol..

[21] Sekine T, Perez-Potti A, Rivera-Ballesteros O, Strålin K, Gorin J-B, Olsson A (2020). Robust T cell immunity in convalescent individuals with asymptomatic or mild COVID-19. Cell.

[22] Yu D, Larsson A, Parke Å, Unge C, Henning C, Sundén-Cullberg J (2020). Single-sampling strategy vs. multi-sampling strategy for blood cultures in sepsis: a prospective non-inferiority study. Front Microbiol..

[23] Szklarczyk D, Gable AL, Nastou KC, Lyon D, Kirsch R, Pyysalo S (2021). The STRING database in 2021: customizable protein-protein networks, and functional characterization of user-uploaded gene/measurement sets. Nucleic Acids Res.

[24] Uhlén M, Fagerberg L, Hallström BM, Lindskog C, Oksvold P, Mardinoglu A (2015). Proteomics. Tissue-based map of the human proteome. Science.

[25] Ritchie ME, Phipson B, Wu D, Hu Y, Law CW, Shi W (2015). limma powers differential expression analyses for RNA-sequencing and microarray studies. Nucleic Acids Res.

[26] Lamers MM, Haagmans BL (2022). SARS-CoV-2 pathogenesis. Nat Rev Microbiol.

[27] Zhang X, Tan Y, Ling Y, Lu G, Liu F, Yi Z (2020). Viral and host factors related to the clinical outcome of COVID-19. Nat Engl.

[28] Zhou F, Yu T, Du R, Fan G, Liu Y, Liu Z (2020). Clinical course and risk factors for mortality of adult inpatients with COVID-19 in Wuhan, China: a retrospective cohort study. Lancet (London, England).

[29] Berlin DA, Gulick RM, Martinez FJ (2020). Severe Covid-19. N Engl J Med United States.

[30] Guarnera A, Podda P, Santini E, Paolantonio P, Laghi A (2021). Differential diagnoses of COVID-19 pneumonia: the current challenge for the radiologist-a pictorial essay. Insights Imaging.

[31] Meyer NJ, Gattinoni L, Calfee CS (2021). Acute respiratory distress syndrome. Lancet (London, England).

[32] Levi M, van der Poll T (2010). Inflammation and coagulation. Crit Care Med United States.

[33] Wool GD, Miller JL (2021). The impact of COVID-19 disease on platelets and coagulation. Pathobiology.

[34] Ishihara J, Ishihara A, Starke RD, Peghaire CR, Smith KE, McKinnon TAJ (2019). The heparin binding domain of von Willebrand factor binds to growth factors and promotes angiogenesis in wound healing. Blood.

[35] Dickneite G, Herwald H, Korte W, Allanore Y, Denton CP, Matucci CM (2015). Coagulation factor XIII: a multifunctional transglutaminase with clinical potential in a range of conditions. Thromb Haemost Germany.

[36] Watanabe-Kusunoki K, Nakazawa D, Ishizu A, Atsumi T (2020). Thrombomodulin as a physiological modulator of intravascular injury. Front Immunol.

[37] Shen D, Podolnikova NP, Yakubenko VP, Ardell CL, Balabiyev A, Ugarova TP (2017). Pleiotrophin, a multifunctional cytokine and growth factor, induces leukocyte responses through the integrin Mac-1. J Biol Chem.

[38] Stern J-B, Paugam C, Validire P, Adle-Biassette H, Jaffré S, Dehoux M (2006). Cytokeratin 19 fragments in patients with acute lung injury: a preliminary observation. Intensive Care Med United States.

[39] Matsumoto K, Nakamura T (1991). Hepatocyte growth factor: molecular structure and implications for a central role in liver regeneration. J Gastroenterol Hepatol Australia.

[40] Michalopoulos GK, DeFrances MC (1997). Liver regeneration. Science United States.

[41] Madonna R, Cevik C, Nasser M, De Caterina R (2012). Hepatocyte growth factor: molecular biomarker and player in cardioprotection and cardiovascular regeneration. Thromb Haemost Germany.

[42] Perreau M, Suffiotti M, Marques-Vidal P, Wiedemann A, Levy Y, Laouénan C (2021). The cytokines HGF and CXCL13 predict the severity and the mortality in COVID-19 patients. Nat Commun.

[43] Wang QS, Edahiro R, Namkoong H, Hasegawa T, Shirai Y, Sonehara K (2022). The whole blood transcriptional regulation landscape in 465 COVID-19 infected samples from Japan COVID-19 Task Force. Nat Commun.

[44] Combes AJ, Courau T, Kuhn NF, Hu KH, Ray A, Chen WS (2021). Global absence and targeting of protective immune states in severe COVID-19. Nature.

[45] Merad M, Blish CA, Sallusto F, Iwasaki A (2022). The immunology and immunopathology of COVID-19. Science United States.

[46] Qin G, Liu S, Yang L, Yu W, Zhang Y (2021). Myeloid cells in COVID-19 microenvironment. Signal Transduct Target Ther.

[47] Keskinidou C, Vassiliou AG, Zacharis A, Jahaj E, Gallos P, Dimopoulou I, et al. Endothelial, immunothrombotic, and inflammatory biomarkers in the risk of mortality in critically ill COVID-19 patients: the role of dexamethasone. Diagnostics (Basel, Switzerland) Switzerland; 2021;11. 10.3390/diagnostics11071249 PMC8304647 34359331

[48] Kooistra EJ, van Berkel M, van Kempen NF, van Latum CRM, Bruse N, Frenzel T (2021). Dexamethasone and tocilizumab treatment considerably reduces the value of C-reactive protein and procalcitonin to detect secondary bacterial infections in COVID-19 patients. Crit Care, England.

[49] Batra R, Whalen W, Alvarez-Mulett S, Gomez-Escobar LG, Hoffman KL, Simmons W (2022). Multi-omic comparative analysis of COVID-19 and bacterial sepsis-induced ARDS. PLoS Pathog.

[50] Leisman DE, Ronner L, Pinotti R, Taylor MD, Sinha P, Calfee CS (2020). Cytokine elevation in severe and critical COVID-19: a rapid systematic review, meta-analysis, and comparison with other inflammatory syndromes. Lancet Respir Med.

[51] Mudd PA, Crawford JC, Turner JS, Souquette A, Reynolds D, Bender D, et al. Distinct inflammatory profiles distinguish COVID-19 from influenza with limited contributions from cytokine storm. Sci Adv. 2020;6. 10.1126/sciadv.abe3024 PMC7725462 33187979

[52] Karaba AH, Zhou W, Hsieh LL, Figueroa A, Massaccesi G, Rothman RE (2022). Differential cytokine signatures of severe acute respiratory syndrome coronavirus 2 (SARS-CoV-2) and influenza infection highlight key differences in pathobiology. Clin Infect Dis an Off Publ Infect Dis Soc Am.

[53] Horby P, Lim WS, Emberson JR, Mafham M, Bell JL, Linsell L (2021). Dexamethasone in hospitalized patients with Covid-19. N Engl J Med.

[54] Shankar-Hari M, Vale CL, Godolphin PJ, Fisher D, Higgins JPT, Spiga F (2021). Association between administration of IL-6 antagonists and mortality among patients hospitalized for COVID-19: a meta-analysis. JAMA.

[55] Baricitinib in patients admitted to hospital with COVID-19 (RECOVERY): a randomised, controlled, open-label, platform trial and updated meta-analysis. Lancet (London, England). 2022;400:359–68. 10.1016/S0140-6736(22)01109-6 PMC9333998 35908569

[56] Nikolla DA, Forehand BR. Do corticosteroids reduce mortality or progression to severe disease for non-oxygen requiring patients infected With COVID-19? Ann Emerg Med. 2022. 10.1016/j.annemergmed.2022.02.005 PMC9021012 35461721

[57] Marshall JC (2014). Why have clinical trials in sepsis failed?. Trends Mol Med England.

[58] Cohen J, Carlet J (1996). INTERSEPT: an international, multicenter, placebo-controlled trial of monoclonal antibody to human tumor necrosis factor-alpha in patients with sepsis. International Sepsis Trial Study Group. Crit Care Med United States.

[59] Fisher CJJ, Agosti JM, Opal SM, Lowry SF, Balk RA, Sadoff JC (1996). Treatment of septic shock with the tumor necrosis factor receptor: Fc fusion protein. The Soluble TNF Receptor Sepsis Study Group. N Engl J Med United States.

[60] Antcliffe DB, Burnham KL, Al-Beidh F, Santhakumaran S, Brett SJ, Hinds CJ (2019). Transcriptomic signatures in sepsis and a differential response to steroids. From the VANISH randomized trial. Am J Respir Crit Care Med.

[61] Meisel C, Schefold JC, Pschowski R, Baumann T, Hetzger K, Gregor J (2009). Granulocyte-macrophage colony-stimulating factor to reverse sepsis-associated immunosuppression: a double-blind, randomized, placebo-controlled multicenter trial. Am J Respir Crit Care Med United States.

[62] Payen D, Faivre V, Miatello J, Leentjens J, Brumpt C, Tissières P (2019). Multicentric experience with interferon gamma therapy in sepsis induced immunosuppression. A case series. BMC Infect Dis.

[63] van Laarhoven A, Kurver L, Overheul GJ, Kooistra EJ, Abdo WF, van Crevel R (2021). Interferon gamma immunotherapy in five critically ill COVID-19 patients with impaired cellular immunity: a case series. Med (New York, NY).

