# Supplementary material for: Targeted plasma proteomics reveals signatures discriminating COVID-19 from sepsis with pneumonia
Source: Respir Res. 2023 Feb 24;24:62. doi: 10.1186/s12931-023-02364-y (PMC9950694; doi:10.1186/s12931-023-02364-y)
Supplement: Supplementary file 1 — Additional file 1: Figure S1. Boxplots of plasma protein levels measured through Proximity Extension Assay. Figure S2. Comparison of protein levels between COVID-19 and Sepsis including adjustment for confounders. Figure S3. Schematic depiction of the machine learning algorithms used to identify biomarkers for differentiating COVID-19 from CAP-Sepsis. Figure S4. Complementary results from machine learning algorithms. Figure S5. Comparative analysis of changes in proteins levels occurring in COVID-19 during acute and convalescence phase. Figure S6. Heatmap showing correlations between the 47 differentially altered plasma proteins in severe COVID-19 and clinical biomarkers. Figure S7. Boxplots of reanalysis of publicly available data provided by Filbin et al. (2021). of the 42 proteins related to COVID-19 severity in our study. Figure S8. Heatmaps of correlations between the 47 proteins related to severity of COVID-19 and cellular markers of monocytes and granulocytes. The Karolinska KI/K COVID-19 Study Group [file 12931_2023_2364_MOESM1_ESM.pdf]

Supporting Information for

Targeted plasma proteomics reveals signatures discriminating COVID-19 from sepsis with pneumonia

Laura M. Palma Medina*[,1], Haris Babačić*[,2], Majda Dzidic[1], Åsa Parke[3,4], Marina Garcia[1], Kimia Maleki[1], Christian Unge[3,5], Magda Lourda[1,6], Egle Kvedaraite[1,7], Puran Chen[1], Jagadeeswara Rao Muvva[1], Martin Cornillet[1], Johanna Emgård[1], Kirsten Moll[1], Karolinska KI/K COVID-19 Study Group, Jakob Michaëlsson[1], Malin Flodström-Tullberg[1], Susanna Brighenti[1], Marcus Buggert[1], Jenny Mjösberg[1], Karl-Johan Malmberg[1], Johan K. Sandberg[1], Sara Gredmark-Russ[1,4,8], Olav Rooyackers[9,10], Mattias Svensson[1], Benedict J. Chambers[1], Lars I. Eriksson, Maria Pernemalm[2], Niklas K. Björkström[1], Soo Aleman[3,4], Hans-Gustaf Ljunggren[1], Jonas Klingström[1], Kristoffer Strålin[3,4,+], Anna Norrby-Teglund[1,+]

Corresponding authors:
Anna Norrby-Teglund, email: anna.norrby-teglund@ki.se
Laura Palma Medina, email: Laura.palma.medina@ki.se

This PDF file includes:

- Figure S1. Boxplots of plasma protein levels measured through Proximity Extension Assay.
- Figure S2. Comparison of protein levels between COVID-19 and Sepsis including adjustment for confounders.
- Figure S3. Schematic depiction of the machine learning algorithms used to identify biomarkers for differentiating COVID-19 from CAP-Sepsis.
- Figure S4. Complementary results from machine learning algorithms.
- Figure S5. Comparative analysis of changes in proteins levels occurring in COVID-19 during acute and convalescence phase.
- Figure S6. Heatmap showing correlations between the 47 differentially altered plasma proteins in severe COVID-19 and clinical biomarkers.
- Figure S7. Boxplots of reanalysis of publicly available data provided by Filbin et al. (2021). of the 42 proteins related to COVID-19 severity in our study.
- Figure S8. Heatmaps of correlations between the 47 proteins related to severity of COVID-19 and cellular markers of monocytes and granulocytes.
- The Karolinska KI/K COVID-19 Study Group

Additional supporting information attached as Excel file with the manuscript:

- Table S1. Statistical comparisons of clinical data.
- Table S2. Statistical comparisons of patient characteristics.
- Table S3. NPX values of proteins measured by PEA.
- Table S4. Statistical comparisons of protein plasma levels between the cohorts and healthy.
- Table S5. Statistical comparison and fold change of protein levels between COVID-19, all CAP and other Sepsis groups.
- Table S6. Coefficients of limma model comparing protein levels of COVID-19 and Sepsis cohorts adjusting for confounders.
- Table S7. Levels of plasma proteins part of the coagulation cascade.
- Table S8. Statistical comparisons of protein plasma levels in the coagulation cascade between all cohorts.

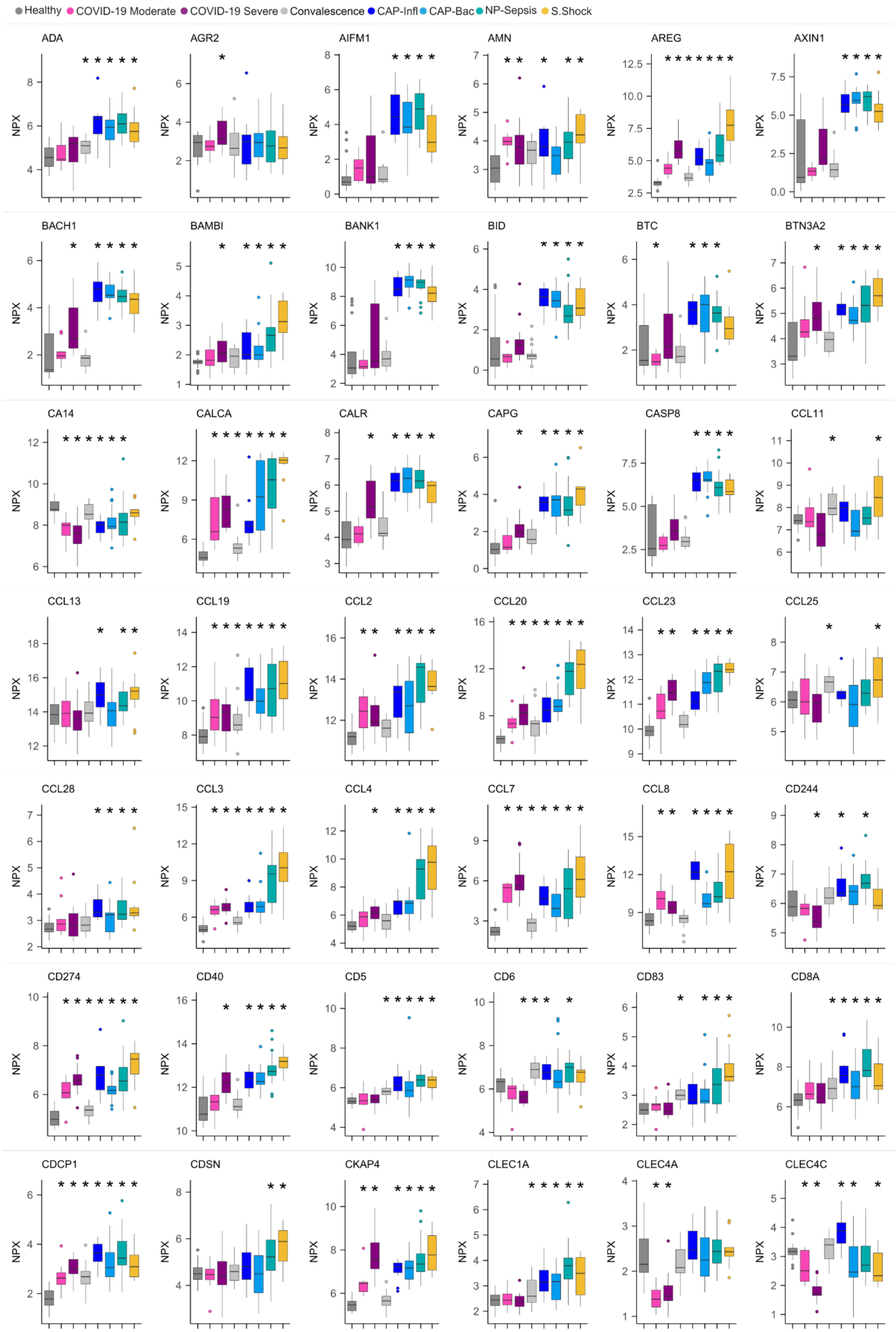

Continue in the next page

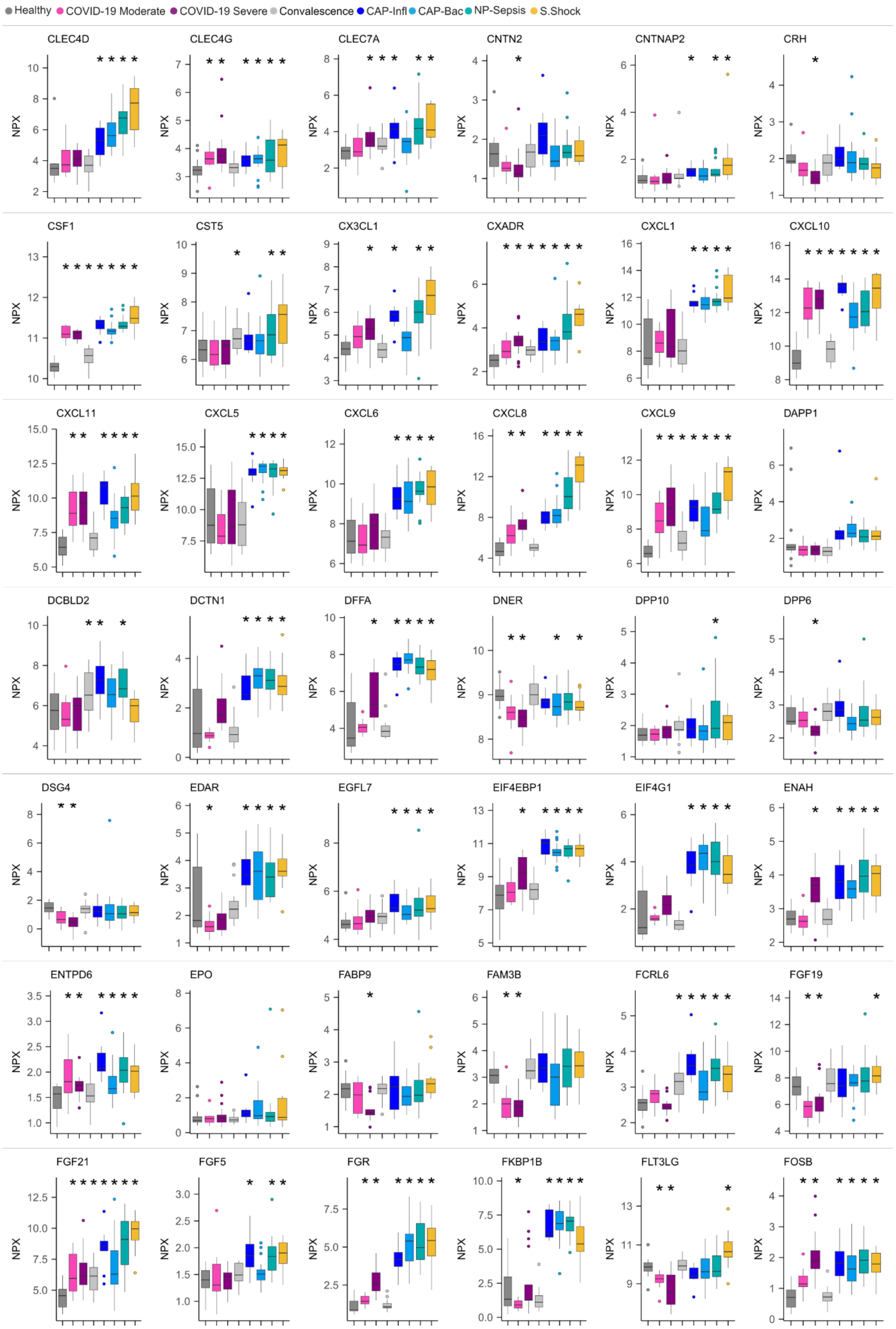

Continue in the next page

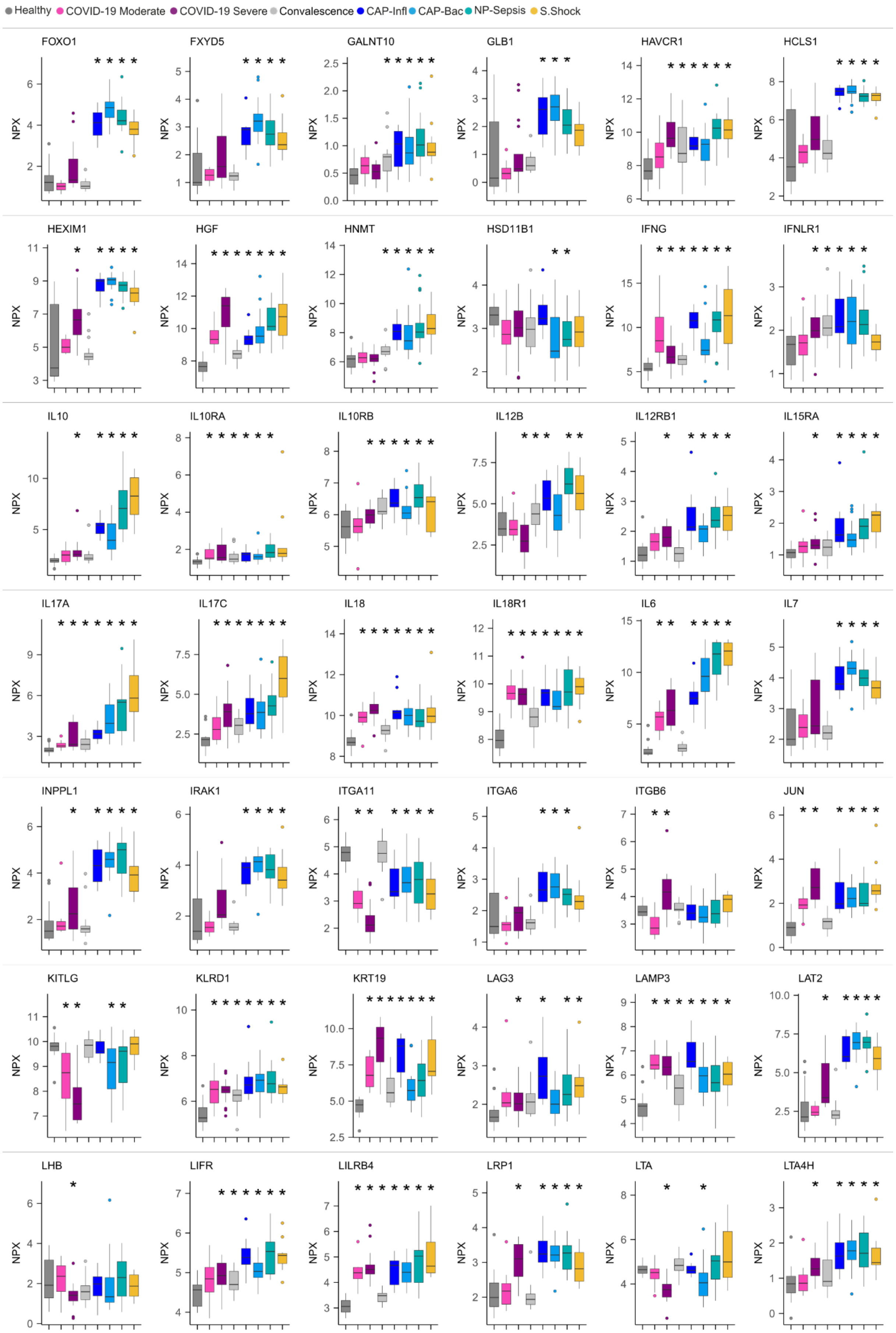

Continue in the next page

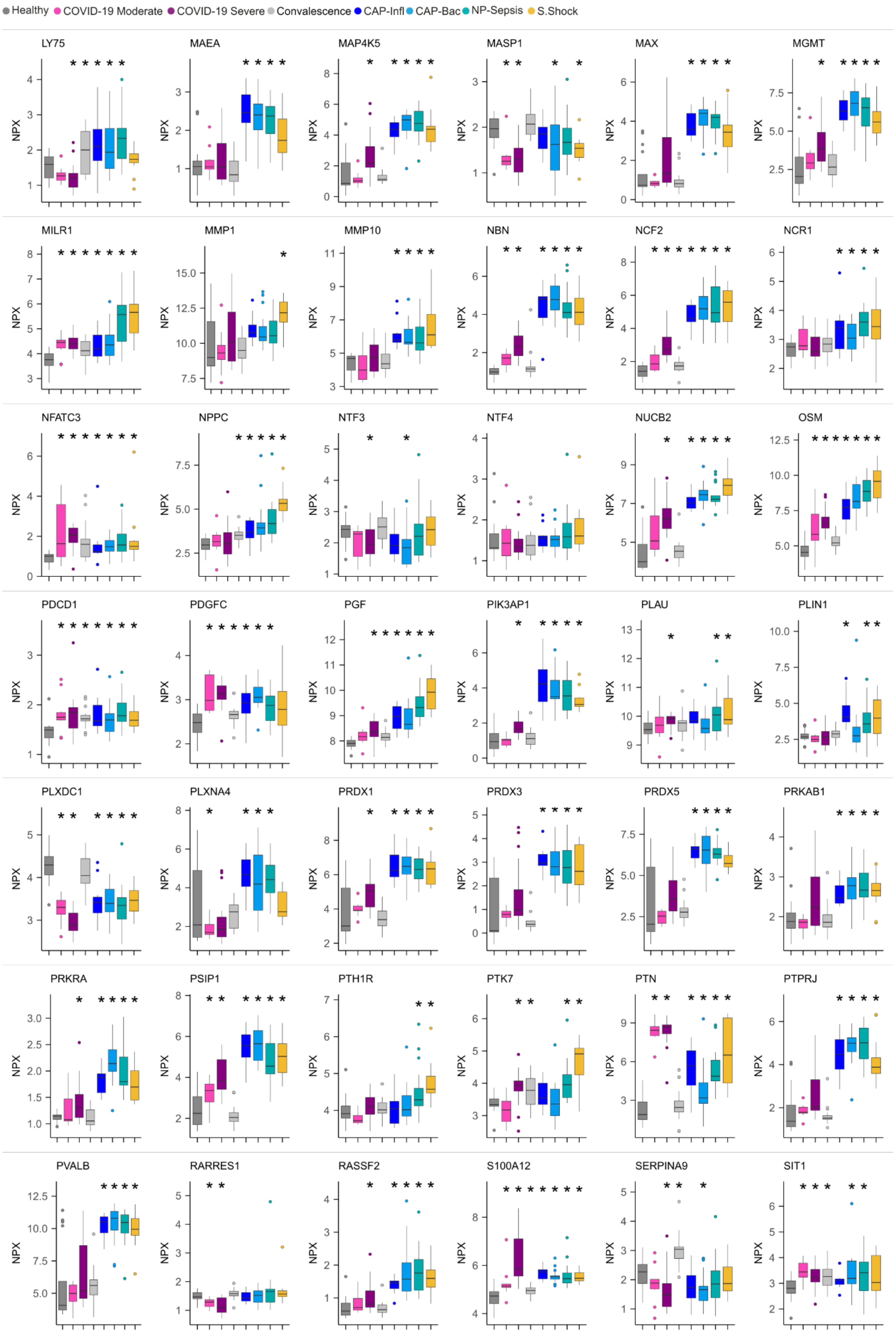

Continue in the next page

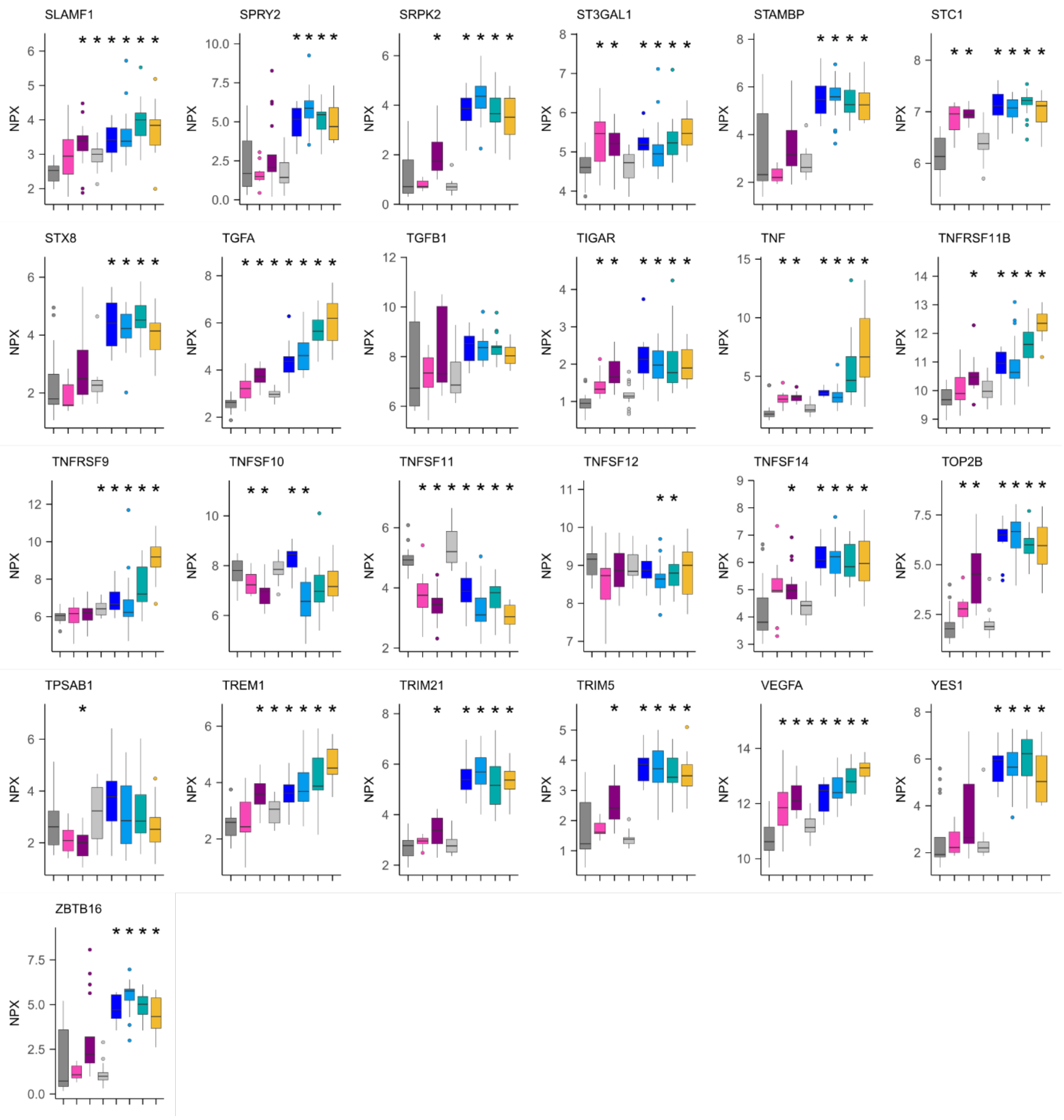

Figure S1. Boxplots of plasma protein levels measured through Proximity Extension Assay. Only the proteins with at least 66% of all samples measured are shown. Patient subgroups are differentiated by color. A star represents significance in comparison to healthy controls q-value<0.05. The boxplots are labeled with gene names.

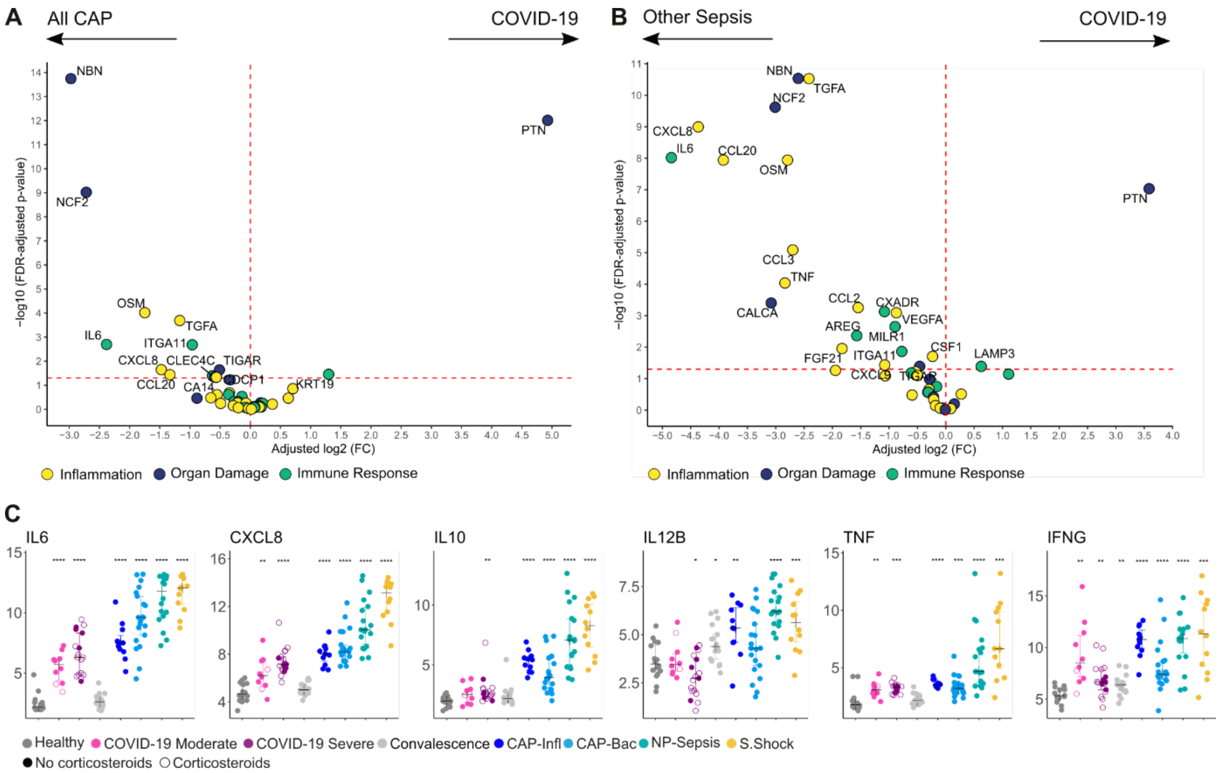

Figure S2. Comparison of protein levels between COVID-19 and Sepsis including adjustment for confounders. A-B. Volcano plots of the difference in adjusted plasma levels of the Core-Pneumonia (A) and Core-other sepsis protein sets (B) as explained in Figure 2H-I in the main manuscript. The adjustment is based on a limma model including age, sex, Charlson comorbidity index and use of corticosteroids prior to sampling as covariates. The proteins are color-coded based on the PEA panel. The horizontal dashed line indicates adjusted p-values= 0.05; **C.** Plasma levels of classical sepsis-associated cytokines as in Figure 2C. The open symbols indicate corticosteroid use prior sampling.

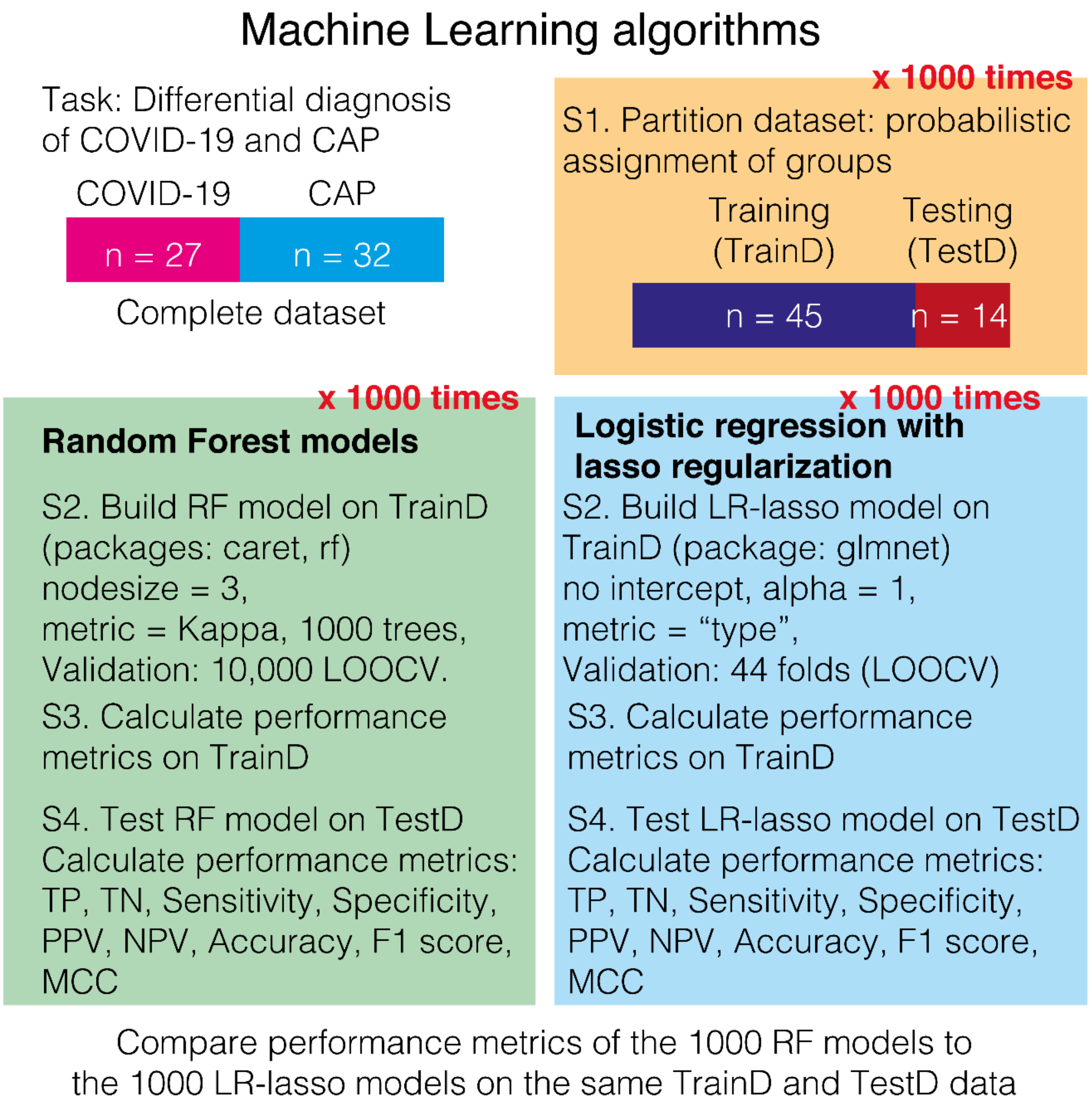

Figure S3. Schematic depiction of the machine learning algorithms used to identify biomarkers for differentiating COVID-19 from CAP-sepsis. Abbreviations: RF = random forest, LR-lasso = logistic regression with lasso regularization; LOOCV = leave-one-out cross validation; TP = true positives; TN = true negatives; PPV = positive predictive value; NPV = negative predictive value; MCC = Mathew's correlation coefficient.

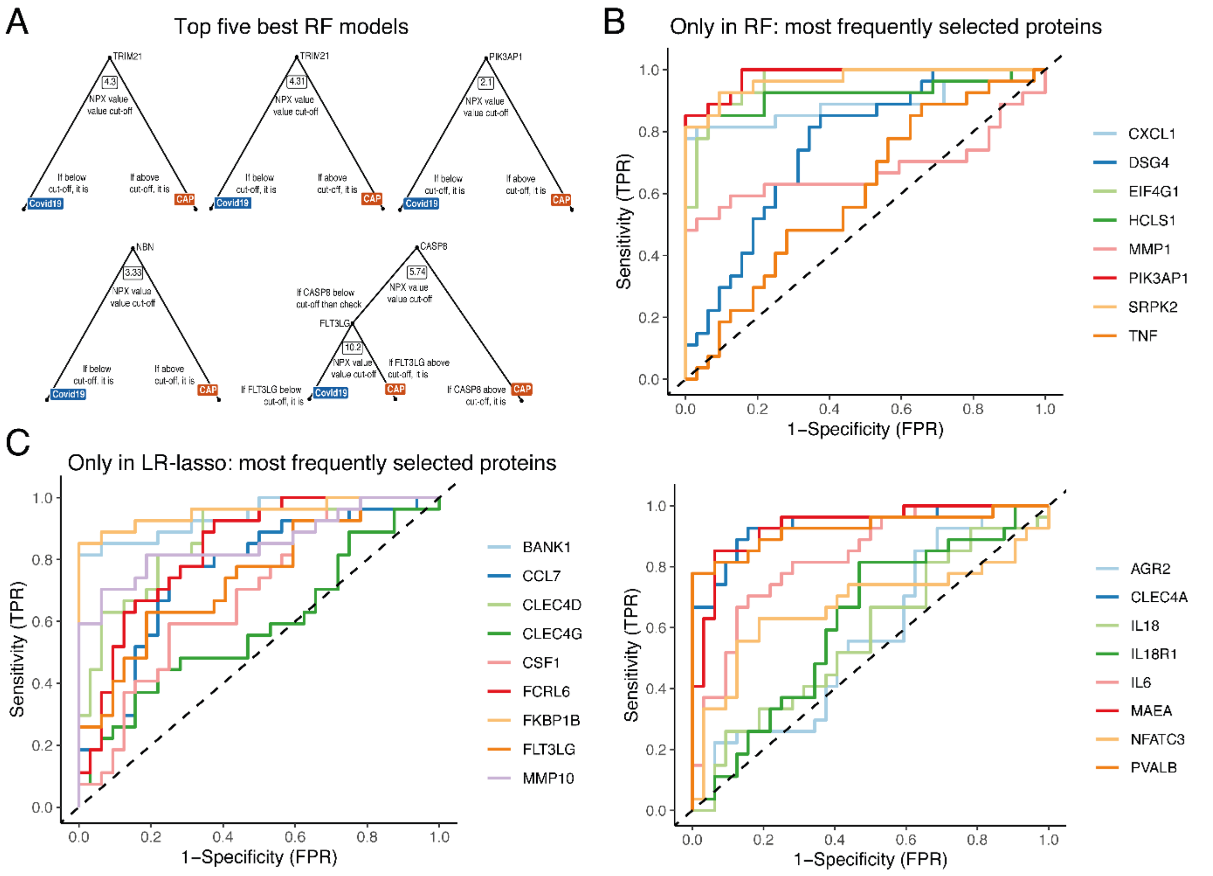

Figure S4. Complementary results from machine learning algorithms. A. Decision trees for the top five random forest (RF) models with highest accuracy on training and testing data (98% and 100%, respectively); B. ROC curves for proteins identified more frequently only in RF; C. ROC curves for proteins identified more frequently only in Logistic regression with lasso regularization (LR-lasso) models. Proteins included in both graphs (left and right) were selected based on display convenience.

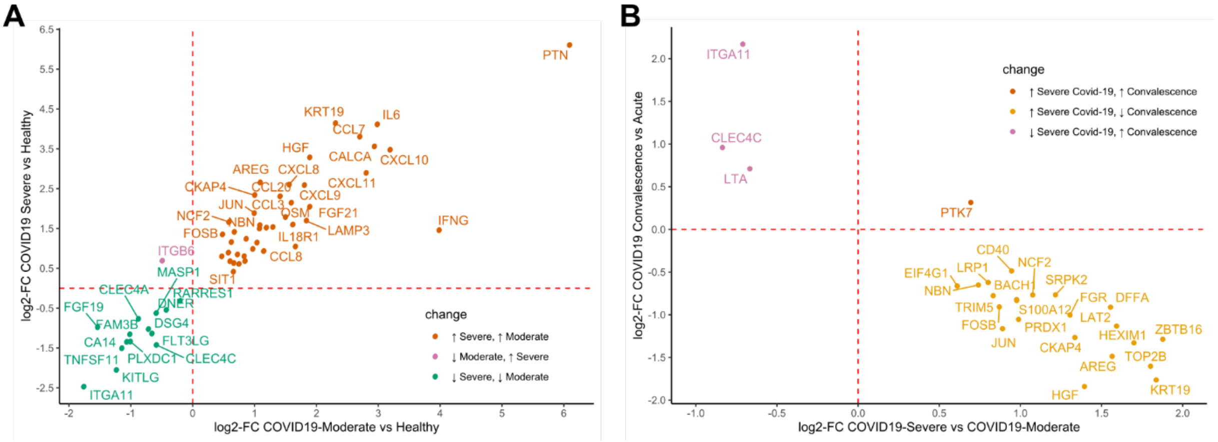

Figure S5. Comparative analysis of changes in protein levels occurring in COVID-19 during acute and convalescence phase. A. Agreement plot comparing the log2-FC of plasma proteome alterations in Moderate COVID-19 (x axis) to the log2-FC of plasma proteome alterations in Severe COVID-19 (y axis), as compared to healthy controls (t-test, $p < 0.05$, 5% FDR); B. Agreement plot comparing the log2-FC of plasma proteome alterations in the comparison Severe to Moderate COVID-19 (x axis, t-test, $p < 0.05$, 5% FDR) to the log2-FC of plasma proteome alterations in matched convalescent and acute COVID-19 samples (paired t test, $p < 0.05$, 5% FDR);

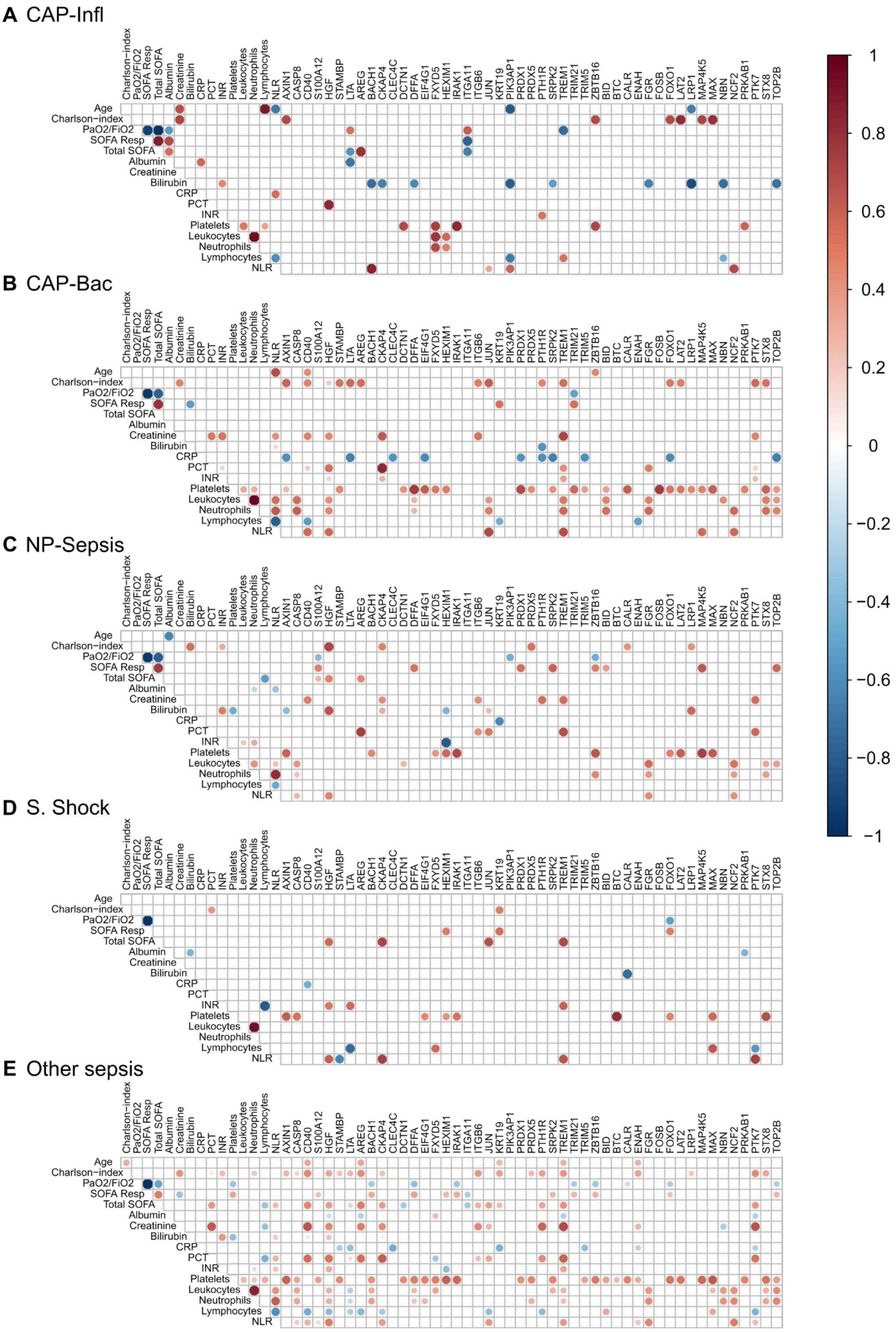

Figure S6. Heatmap showing correlations between the 47 differentially altered plasma proteins in severe COVID-19 and clinical biomarkers. Each panel shows the correlations made with samples from each cohort of patients. Only correlations that are statistically significant (Spearman's ρ, $p < 0.05$) are plotted. The bigger circle size and higher colour intensity represent more significant correlations.

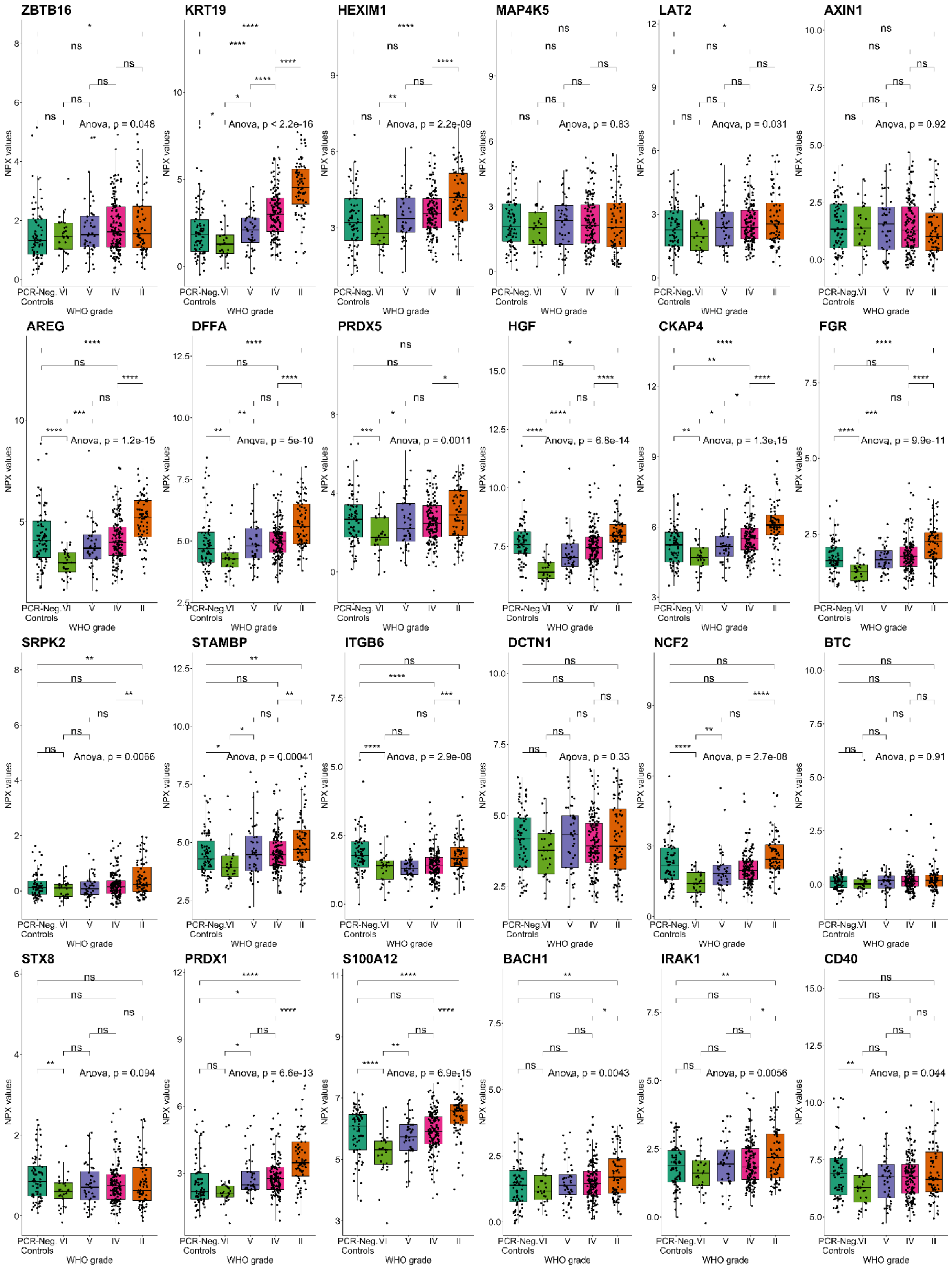

Continues in the next page

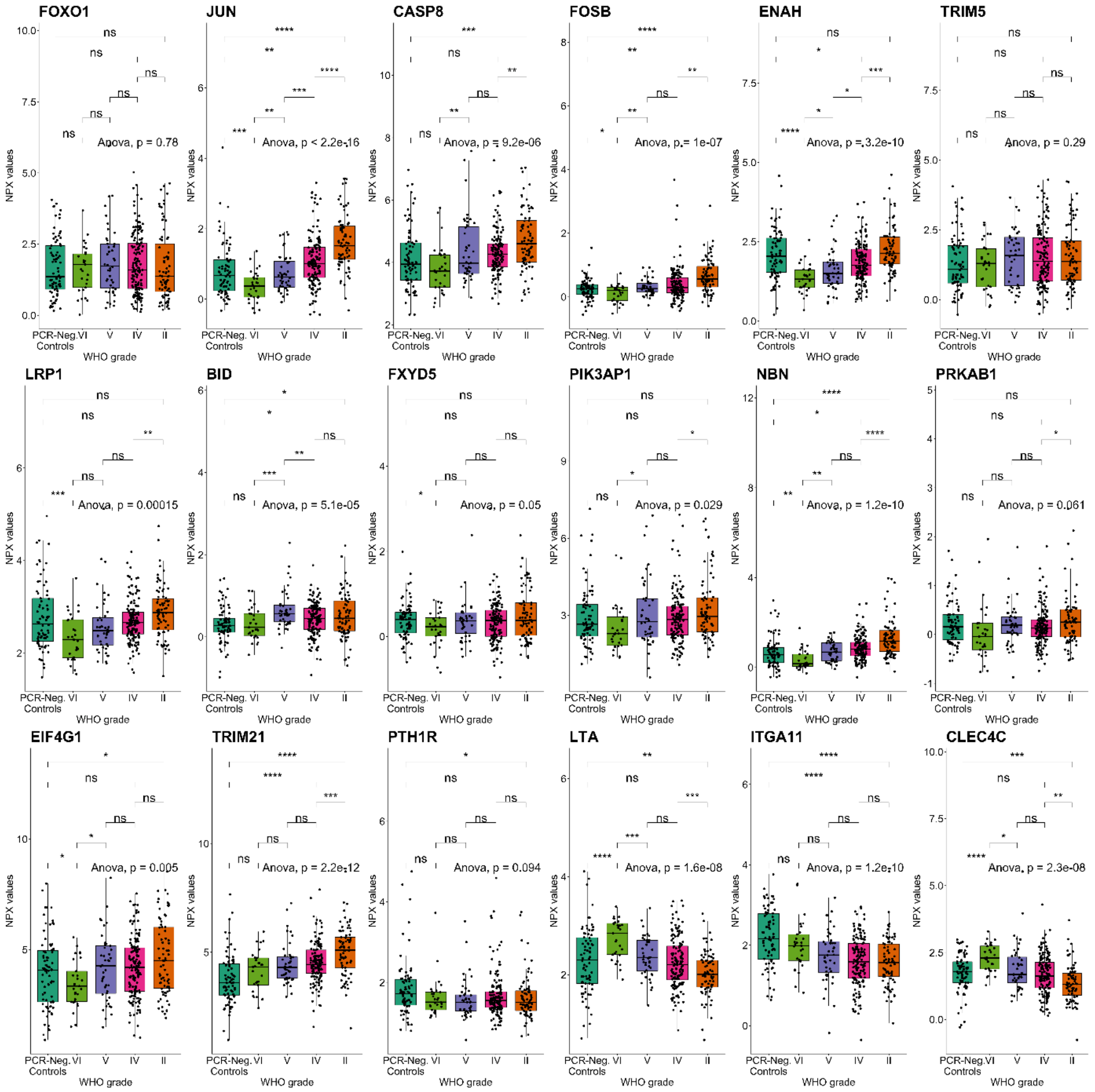

Figure S7 Boxplots comparing the NPX values of the 42 proteins identified in our study to be different between severe and moderate COVID-19 - reanalysis of publicly available data provided by Filbin et al. (2021). Different WHO grades of COVID-19 severity at day 0 (admission to hospital) are plotted on the x axis, in comparison to PCR-negative hospital controls. Only 42 proteins of the 47 identified in our study are shown as the data for the remaining 5 was not available in Filbin et. al. The grades II and IV are comparable to our classification of severe and moderate, respectively. The ANOVA p value refers to the variance across the groups, whereas the stars represent statistical significance determined with a t test, as follows - ns: p > 0.05, *: p <= 0.05, **: p <= 0.01, ***: p <= 0.001, ****: p <= 0.0001.

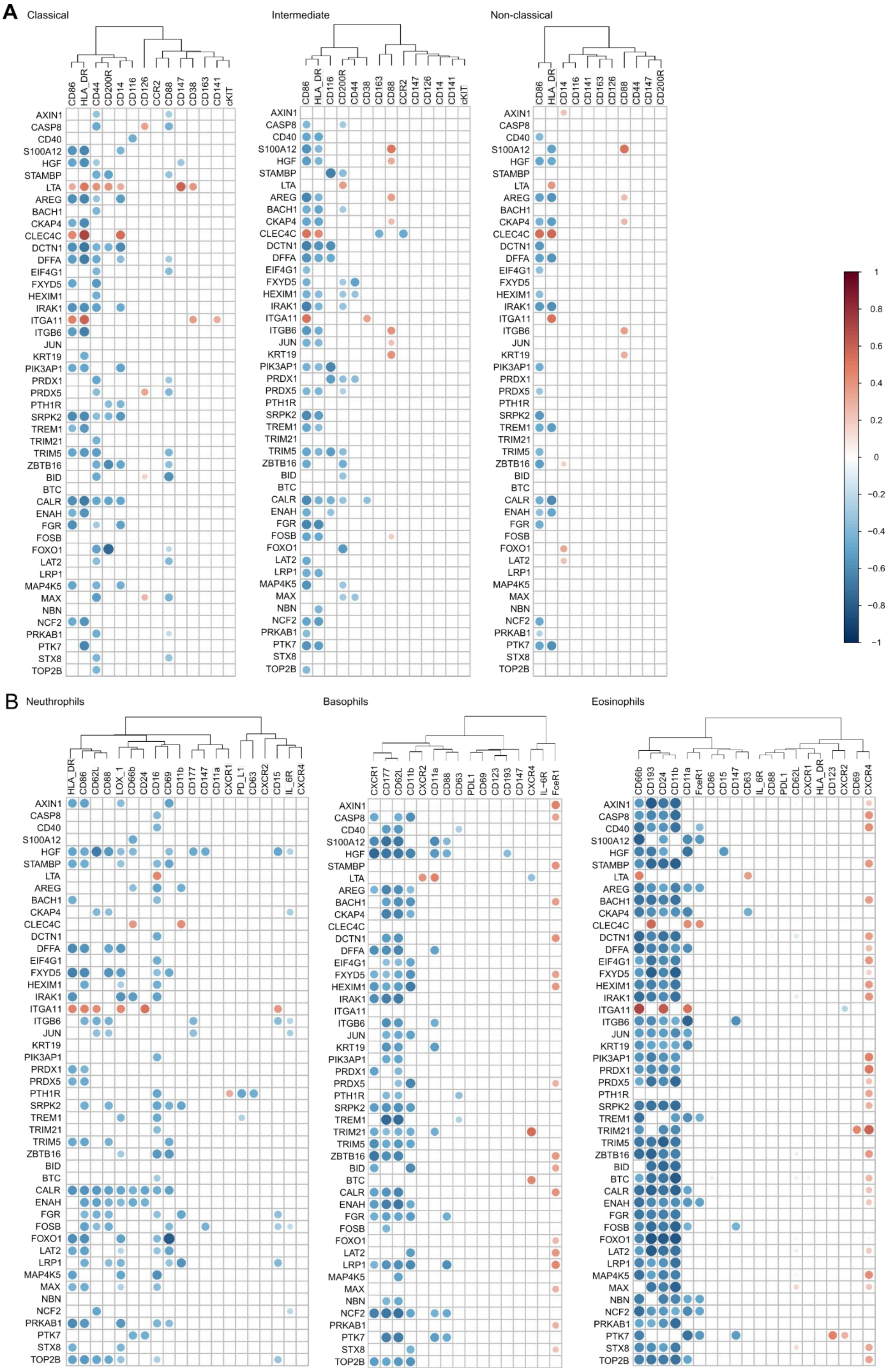

Figure S8. Heatmaps of correlations between the 47 proteins related to severity of COVID-19 and median fluorescence intensity (MFI) of extracellular receptors on monocytes and granulocyte subsets. A. MFI of surface protein markers identifying and characterizing monocyte subsets. B. MFI of surface protein markers identifying and characterizing neutrophils, basophils and eosinophils.

The Karolinska COVID-19 Study Group

Mira Akber[1]; Soo Aleman[2,3]; Lena Berglin[1]; Helena Bergsten[1]; Niklas K Björkström[1]; Susanna Brighenti[1]; Demi Brownlie[1]; Marcus Buggert[1]; Marta Butrym[1]; Benedict J Chambers[1]; Puran Chen[1]; Martin Cornillet[1]; Angelica Cuapio[1]; Isabel Diaz Lozano[1]; Lena Dillner[3]; Therese Djärv[4]; Majda Dzidic[1]; Johanna Emgård[1]; Lars I Eriksson[5]; Malin Flodström-Tullberg[1]; Hedvig Glans[3]; Jean-Baptiste Gorin[1]; Sara Gredmark-Russ[1,3,6]; Jonathan Grip[5]; Quirin Hammer[1]; Alvaro Haroun-Izquierdo[1]; Elisabeth Henriksson[1]; Laura Hertwig[1]; Sadaf Kalsum[1]; Tobias Kammann[1]; Jonas Klingström[1]; Efthymia Kokkinou[1]; Egle Kvedaraite[1,7]; Hans-Gustaf Ljunggren[1]; Marco Giulio Loreti[1]; Magdalini Lourda[1,8]; Kimia T Maleki[1]; Karl-Johan Malmberg[1]; Nicole Marquardt[1]; Johan Mårtensson[5]; Christopher Maucourant[1]; Jakob Michaëlsson[1]; Jenny Mjösberg[1]; Kirsten Moll[1]; Jagadeeswara Rao Muvva[1]; Pontus Nauclér[3]; Anna Norrby-Teglund[1]; Laura M. Palma Medina[1]; Tiphaine Parrot[1]; André Perez-Potti[1]; Björn P Persson[5]; Lena Radler[1]; Dorota Religa[9]; Emma Ringqvist[1]; Olga Rivera-Ballesteros[1]; Olav Rooyackers[5,10]; Johan K Sandberg[1]; John Tyler Sandberg[1]; Takuya Sekine[1]; Ebba Sohlberg[1]; Tea Soini[1]; Anders Sönnerborg[2,3]; Kristoffer Strålin[2,3]; Benedikt Strunz[1]; Mattias Svensson[1]; Janne Tynell[1]; Christian Unge[2,4]; Renata Varnaite[1]; Andreas von Kries[1]; David Wullimann[1]

Affiliations:

[1]Center for Infectious Medicine, Department of Medicine Huddinge, Karolinska Institutet, Karolinska University Hospital, Stockholm, Sweden

[2]Department of Medicine Huddinge, Karolinska Institute, Stockholm, Sweden

[3]Department of Infectious Diseases, Karolinska University Hospital, Stockholm, Sweden

[4]Department of Emergency Medicine, Karolinska University Hospital, Stockholm, Sweden

[5]Department of Perioperative Medicine and Intensive Care, Karolinska University Hospital, Stockholm, Sweden

[6]The Laboratory for Molecular Infection Medicine Sweden (MIMS), Umeå, Sweden

[7]Department of Clinical Pathology and Cancer Diagnostics, Karolinska University Hospital, Stockholm, Sweden

[8]Childhood Cancer Research Unit, Department of Women's and Children's Health, Karolinska Institutet, Stockholm, 171 77 Sweden

[9]Theme Aging, Karolinska University Hospital, Stockholm, Sweden

[10]Department of Clinical Interventions and Technology CLINTEC, Division for Anesthesiology and Intensive Care, Karolinska Institutet, Stockholm, Sweden.
